# The effects of clinical supervision on supervisees and patient outcomes in psychotherapy – a systematic review and meta-analysis

**DOI:** 10.3389/fpsyt.2025.1705578

**Published:** 2025-11-21

**Authors:** Bianca Schreyer, Cosima Leithner, Rebekka Eilers, Katharina Gossmann, Rita Rosner

**Affiliations:** 1 Department of Psychology, Faculty of Philosophy and Education, Catholic University Eichstaett-Ingolstadt, Eichstätt, Germany; 2 Department of Psychology, Faculty of Psychology and Educational Sciences, Ludwig-Maximilians-University Munich, Munich, Germany

**Keywords:** supervision, psychotherapy, therapists in training, therapist competence, therapeutic alliance, patient symptoms, meta-analysis

## Abstract

**Objective:**

Supervisions and case consultations are an essential and often required part of psychotherapeutic training. Despite its importance and ubiquitous use, research is limited, and no quantitative review is yet available. This systematic review and meta-analysis provides an overview of empirical literature on clinical supervision and investigates its effectiveness regarding various outcomes (PROSPERO CRD42024574318).

**Method:**

We conducted a comprehensive literature search up to June 16^th^, 2025, in the databases Epistemonikos, Cochrane Library, EMBASE, PubMed, PsycINFO and Web of Science. Eligible trials were controlled and uncontrolled trials evaluating the effectiveness of clinical supervision/case consultation with pre- and post-assessment points. Study quality was assessed using Cochrane’s ROBINS-I V2 and RoB-2 tool. Where applicable, outcomes were analyzed using a random-effects model meta-analysis.

**Results:**

32 studies met the inclusion criteria of which 13 studies could be included in meta-analyses for the outcomes on therapists’ competence, therapeutic alliance and patients’ symptoms. Supervision interventions yielded a small but non-significant effect on symptom reduction in patients favoring the respective supervision interventions. There was a small effect on therapists’ competence and medium effect on therapeutic alliance, which both were non-significant, yet large and significant when compared with passive controls. Regarding the studies included in the review, supervision was mostly reported to have a beneficial effect on therapists’ competence, alliance and patients’ symptoms, while the effects on therapists’ adherence were heterogeneous.

**Conclusion:**

These results highlight promising effects of supervision and the need for further investigation of different supervision models. Further research is needed to examine the effectiveness of supervision and investigate potential moderating factors.

**Systematic Review Registration:**

https://www.crd.york.ac.uk/prospero/, identifier CRD42024574318.

## Background

Clinical supervisions and case consultations are mandatory parts of psychotherapeutic training in various countries. It is also considered as one of the key elements of professional development for therapists (1, 2). Yet, there are multiple definitions of clinical supervision. In the context of this review we will refer to clinical supervision as supervision and understand it as a “working alliance between the supervisor and counsellor in which the counsellor can offer an account or recording of her work; reflect on it; receive feedback and where appropriate, guidance” (3 as cited in 4, p. 54). In this review we adjust the cited definition by including next to counsellors also various groups of psychotherapeutically working professionals like psychotherapists, psychiatrists, psychologists, medical practitioners, and social workers. In the following, the term supervision model refers to the general theoretical framework and rational of supervision, while supervision intervention describes the implementation of supervision practices, such as duration, frequency and setting. In general, the supervisor is a professionally approved senior (2). Supervision fulfils three main functions: quality control, the maintenance and development of professional competencies, and the facilitation of effective practice (5). These functions integrate the elements of skill development and reflective practice to uphold both therapeutic standards and professional development. While supervision is generally considered helpful for the supervisees themselves (6), the “acid test” of good supervision is considered to be the improvement of patients’ symptoms through supervision (7, p. 485). However, empirical evidence on supervision’s impact is inconsistent and studies report heterogeneous effects on the supervisees and their patients, highlighting the need for further systematic investigation (1, 8).

So far, multiple reviews on supervision have been conducted (e.g. 4, 7–12, 14–18). Wheeler and Richards (4) provide a comprehensive narrative review of qualitative and quantitative studies including surveys and intervention studies. The included studies mainly focused on trainees and short-term outcomes and report usefulness of supervision for the supervisees. Yet the impact of these effects on the supervisees’ patients remained unclear. Alfonsson et al. (8) specifically focused on supervision for psychotherapists providing cognitive behavioral therapy (CBT) in their review. They initially planned on computing a meta-analysis and calculated effect sizes, but did not pool them since too few studies reported identical outcomes. The authors emphasize that although supervision shows positive effects, there are only a few rigorous empirical studies on this topic. The superiority of a specific supervision model could not be identified. Alfonsson et al. (8) criticize the general consensus that supervision is effective while there is a lack of empirical support. Kühne et al. (11) highlight that supervision appears to be beneficial for supervisees, but also emphasize the need for more systematic and methodologically robust research in this field. Keum and Wang (10) as well as Park et al. (15) conducted meta-analyses on the correlation of outcomes associated with supervision and psychotherapy. Keum and Wang (10) reported a small significant correlation between supervision process variables and psychotherapy process and outcome variables, yet these varied largely between the individual studies. Park et al. (15) reported positive correlations between supervisory working alliance and other supervision outcome variables, such as self-efficacy or self-disclosure. Another recent review by Lohani and Sharma (12) emphasizes that supervision positively affects supervisees’ self-awareness and self-efficacy.

Vezer (16) and Maaß et al. (17) both conducted more recent reviews on the literature focusing on live supervision as a specific supervision technique. In this approach, the supervisor observes the supervisee during therapy sessions and provides real-time feedback on the therapist’s behavior. This is often implemented through bug-in-the-eye (BITE) supervision, where the therapist is provided with written real-time feedback via a monitor that is only visible to them. Alternatively, live supervision can also be conducted using audio-feedback. Both reviews identified studies supporting the use of live supervision, especially in the context of CBT, where live supervision was found to be more effective than passive controls and equally as effective as other supervision conditions (16, 17). Most recent reviews all focused on a specific subgroup of supervision (CBT or live supervision). None of the recent meta-analyses conducted an analysis on the effectiveness of supervision only looking at intervention studies and estimated a between-group effect size.

To the best of our knowledge, there is no review that used meta-analysis for estimating the overall effectiveness of supervision on various outcomes regarding the supervisees themselves and their patients. Therefore, the aim of the present study is to provide a comprehensive review of the existing and since the last reviews newly added studies on the effects of supervision on the supervisees themselves and their patients’ symptom-related outcomes. The research questions are: (1) Does supervision for psychotherapists lead to an improvement in therapist characteristics (e.g. increase in knowledge, skills, and self-efficacy)? (2) Does supervision for psychotherapists lead to an improvement in the treatment outcome of their patients? (3) Are there characteristics of supervision (e.g. setting, frequency) that moderate its effectiveness?

## Method

The meta-analysis was registered in PROSPERO (CRD42024574318) and is reported according to the Preferred Reporting Items for Systematic Reviews and Meta-Analysis guideline (PRISMA; 19).

### Search strategy and study selection

The following databases were systematically searched up to June 16^th^ 2025: Epistemonikos, Cochrane Library, EMBASE, PubMed, PsycINFO and Web of Science. The search strategy consisted solely of terms referring to supervision/case consultation and the different categories of psychotherapeutically working professionals. We did not include any search terms for specific outcomes, as the report of various outcomes was expected. MESH-Terms were applied where applicable. The search strategy in detail is provided in **Appendix A**. Following the criteria in the AMSTAR checklist (20), one third of the titles and abstracts were screened by two raters (B.S. and C.L.) and after a sufficient agreement was achieved, one third was screened by one rater each. All full texts were screened by two out of three independent raters each (B.S., C.L. and/or J.G.). Any uncertainties were resolved by consulting a co-author (R.E.).

### Eligibility criteria

We included (1) intervention studies with pre- and post-assessment points published in a peer-reviewed journal in English or German reporting quantitative outcome data on the supervisees or the supervisees’ patients related to the supervision intervention. (2) Supervisees needed to be psychotherapeutically working professionals (fully trained or trainees), this may include psychotherapists, psychologists, psychiatrists, medical practitioners, social workers, and counsellors. Supervisees may have any level of competence or psychotherapy training. (3) Psychotherapy should address a behavioral, emotional, cognitive, social or mental health related issue. (4) Psychotherapy may target any population or subgroup. The intervention is eligible if (5) clinical supervision/case consultation is studied. (6) Supervisors conducting the clinical supervision/case consultation may have any level of competence. (7) The clinical supervision/case consultation may be conducted in any mode (individual/group, with/without videos, any frequency, structured/unstructured, disorder specific/general…). (8) The clinical supervision/case consultation must have a focus on patients treated by supervisees. We excluded studies where the supervisees are case managers or lay counsellors or where the intervention was no supervision with focus on the patients treated by supervisees, but was a training, workshop or presentation or meta-supervision. In cases where supervision was combined with specific training, there had to be a separate post-training and pre-supervision measurement of outcomes or a control group that received the same training and no or a different supervision condition.

For the meta-analysis, only controlled trials were eligible as we expected large heterogeneity in study design and therefore multiple confounders in pre-/post-effect sizes.

### Data extraction

Two individuals (B.S. and J.G.) extracted outcome data on study characteristics and characteristics on supervisor, supervisee and patients as well as the supervision and therapy. Inconsistencies were resolved through discussion. All outcomes for the review were extracted narratively and eligible outcomes for the meta-analysis were coded for effect size calculation (i.e. number of participants per arm, the mean score of outcomes in each arm at posttest and the standard deviation of outcomes in each arm at posttest). Intention-to-treat data were used when available. We initially aimed to calculate meta-analyses for supervisees’ competence, therapeutic alliance, fidelity, adherence and patients’ symptom reduction. For studies included in the meta-analysis, if variables relevant for effect size calculation were not presented, authors were contacted for missing information. If data could not be obtained, the study was excluded from the analysis, but not from the systematic review.

### Risk of bias assessment

Risk of bias of all included studies was assessed using the Risk of Bias assessment tool (RoB 2.0; 21) for randomized controlled trials (RCT) and the Risk of Bias In Non-randomized Studies – of Interventions Version 2 (ROBINS-I V2; Version 1 by 22) assessment tool for non-randomized trials. The RoB 2.0 tool utilizes five domains to rate studies for bias: randomization process, deviations from intended interventions, missing outcome data, measurement of the outcome, and selection of the reported result. These domains are used to determine whether a study has a ‘low risk’, ‘some concerns’, or a ‘high risk’ of bias by an algorithm. The ROBINS-I V2 tool uses seven domains: confounding, selection of participants into the study, classification of intervention, deviations from intended intervention, missing data, measurement of the outcome and selection of the reported results.

### Statistical analysis

The meta-analyses were performed in R and RStudio Version 2024.12.0 (23) using the {metafor} package (24). To conduct meta-analyses an adequate number of trials reporting the outcome of interest was required (*k* ≥ 4). Effect sizes were calculated as standardized mean differences (SMD) using Hedges’ *g* to adjust for small sample bias and 95% confidence intervals (CI). Effect sizes were computed by pooling studies according to the random effects model, as considerable heterogeneity between studies was expected. To assess the need for a three-level model, a comparison was made between two-level and three-level models. The three-level model accounted for within-study and between-study heterogeneity, which was relevant due to the inclusion of multiple interventions within studies. Model fit was assessed using likelihood ratio tests. The two-level model was retained for the two outcomes therapists’ competence and patients’ symptoms, while for therapeutic alliance the three-level model was applied due to better fit. The extent of heterogeneity in the effect sizes was examined using Cochran’s *Q* and *I*^2^ statistics (25). As suggested by Deeks et al. (26) *I*^2^ between 0% and 40% was interpreted as might not be important, 30%-60% as moderate, 50%-90% as substantial, and 75%-100% as considerable heterogeneity. Subgroup analyses are computed assuming that all subgroups are sharing a common estimate of the between-study heterogeneity *^2^.* Note that these are recommended for computation only if the meta-analysis for an outcome contains at least *k =* 10 studies or at least *k* = 4 studies per subgroup (27, 28). We decided to calculate exploratory subgroup analyses for a smaller number of studies to facilitate a descriptive comparison of effects regarding different control groups. In addition, since some studies were identified conducting short-term supervision interventions of less than three supervision sessions, we computed an additional sensitivity analysis that excluded these studies. Publication bias was intended to be assessed inspecting the funnel plot and performing Egger’s test of asymmetry (29) only if more than 10 studies are included in the meta-analysis (30).

To provide a tabular overview of the quality of evidence regarding the effectiveness of supervision we assessed the certainty of evidence for outcomes included in meta-analyses and produced a summary of findings table according to the GRADE working group using GRADEpro (31, 32).

## Results

### Study selection

**Figure 1:**
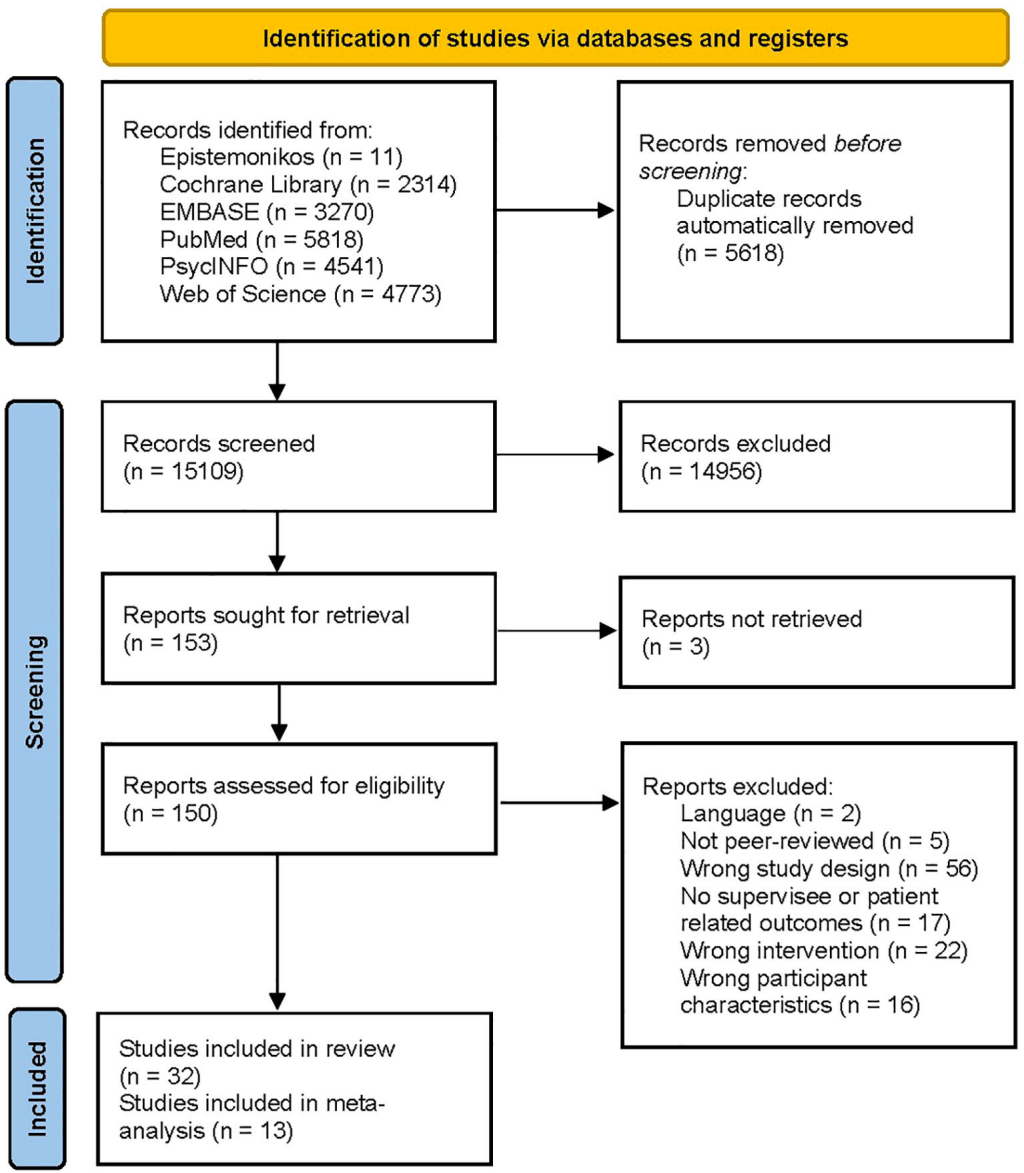
PRISMA flow diagram of study selection process.

An overview of the study selection is shown in a PRISMA flowchart presented in **Figure 1**. Searches produced a total of 20,727 results. After automatic removal of duplicates 15,109 titles and abstracts were screened. 153 studies were sought for retrieval, of which three could not be retrieved. Accordingly, 150 studies were assessed for eligibility through full text screening. In total, 32 studies met the eligibility criteria and were included in the review. The exact citations of these studies are listed in **Appendix B**. Of the outcomes considered, only therapists’ competence, therapeutic alliance, and patients’ symptoms met the criterion for inclusion in meta-analyses (*k* ≥ 4). Accordingly, 13 studies could be included in a meta-analysis. A list of potentially relevant studies that were read in full-text form but excluded can be found in **Appendix C**.

### Characteristics of included studies

Of the 32 studies included, 21 were controlled and 16 RCTs. 12 studies compared the examined supervision intervention to another supervision intervention (mostly supervision-as-usual, SAU, *n* = 4 studies) and nine compared it to passive control conditions. In five studies there was an additional active comparison condition (AC). ACs oftentimes differed from the intervention condition more in terms of technical aspects (e.g. BITE, video-based, audio-based, …) rather than conceptual aspects (e. g. process-focus vs. skill-focus as in 33). In total, data of 1614 supervisees (reported by 32 studies), 2282 patients (reported by 21 studies) and 242 supervisors (reported by 23 studies) were obtained. Supervisees were mostly therapists providing CBT (*n* = 9 studies), followed by Psychodynamic Psychotherapy (*n* = 4 studies), and one study with therapists providing Humanistic Therapy. The remaining studies did not provide information on the theoretical orientation of the supervisees. The supervisee population included various experience levels including fully trained therapists, trainees and mixed populations. Supervisees had a mean age of 32.62 years (*SD* = 8.93), were predominantly female (73.04%) and had a mean experience of 4.84 years (*SD* = 3.08), however the years of experience were reported by 11 studies only. Supervisees’ patients had a mean age of 30.70 (*SD* = 12.72) and were also predominantly female (65.42%). In most studies, there was little detailed information about the supervisors and their qualification/competence level. On average, in active supervision conditions, supervisees received 8.94 supervision sessions (*SD* = 9.61), each lasting *M* = 72.45 minutes (*SD* = 61.36), over a mean period of 13.21 weeks (*SD* = 11.57). Supervisees provided on average 6.28 therapy sessions (*SD* = 5.24) during supervision intervention, each lasting *M* = 32.68 minutes (*SD* = 18.22), over a mean period of 13.21 weeks (*SD =* 11.57). An overview of supervisors’, supervisees’, supervision patients’ and therapy characteristics of the included studies is presented **Appendix D**. An overview of the main outcomes of the included studies is presented in **Appendix E**.

### Risk of bias assessment

Risk of bias ratings for all non-randomized studies was at least serious (*n* = 13 *serious risk*; *n* = 3 *critical risk*), predominantly due to the lack of a pre-registration or published study protocol leading to a serious risk of bias rating in uncontrolled studies with a high potential for reporting bias in uncontrolled designs. Most randomized studies were rated with at least some concerns (*n* = 11 *some concerns*; *n* = 2 *high risk of bias*). The main reason being that the randomization process was not described in detail and the absence of a pre-registration or published study protocol. If the only information about randomization methods is a statement that participants were randomized, the algorithm leads to a *some concerns* rating. Only three studies were rated as *low risk of bias*. A detailed rating per domain for the individual studies can be found in **Appendix F**.

### Effects of supervision on the supervisees’ competence

**Figure 2:**
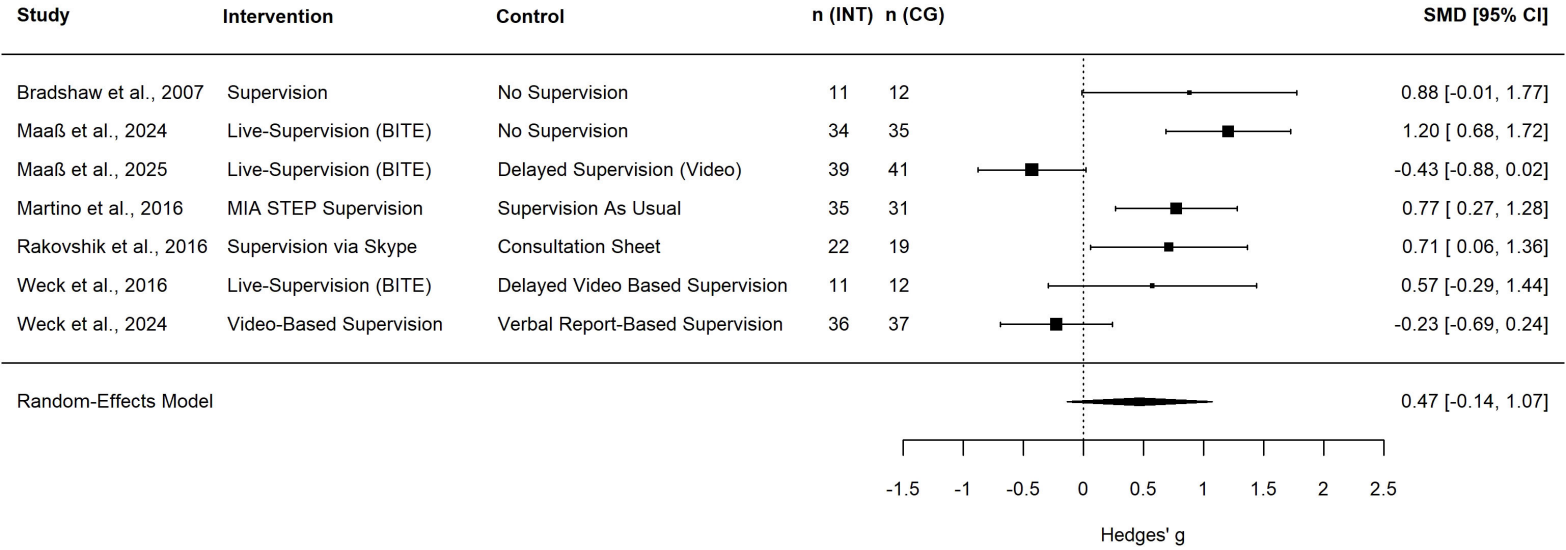
Forest plot of therapists’ competence. Square size indicates study weight; a positive effect size indicates the superiority of the intervention group; n (INT), number of therapists in the intervention group; n (CG), number of therapists in the control group; SMD, standardized mean difference; CI, confidence interval; BITE, bug-in-the-eye supervision; MIA STEP, Motivational Interviewing Assessment Supervisory Tools for Enhancing Proficiency.

In total, 15 studies reported outcomes related to the supervisees’ competence (13, 34–41; 42; 43–47), of which seven trials with four active comparisons and three passive comparisons could be included in the meta-analysis (see **Figure 2** for details). The meta-analysis of these trials found a small non-significant effect of the supervision intervention on supervisees’ competence (*g* = 0.47, 95%-CI [-0.14, 1.07], *k* = 7; see **Table 1** for the meta-analysis and **Figure 2** for the forest plot). Heterogeneity in the meta-analysis was substantial (*I^2^* = 79.1%). When excluding trials with less than three supervision sessions (13, 40, 47) we found a medium and significant effect (*g* = 0.74, 95%-CI [0.20, 1.29], *k* = 4; see **Table 1**). Heterogeneity reduced to zero (*I^2^* = 0%). Due to the observation that most studies used active comparisons that were highly similar to the intervention (e.g., BITE supervision vs. video-based supervision), subgroup analyses were conducted for effect sizes of comparisons with an active or passive control group (**Table 2**). Compared to passive control conditions supervision demonstrated a large and significant effect (*g* = 0.95, 95%-CI [0.14, 1.76], *k* = 3). When compared with ACs no significant effect was observed (*g* = 0.12, 95%-CI [-0.54, 0.78], *k* = 4). The subgroup analysis was non-significant (*p* = 0.09).

**Table 1:** Between-group effect sizes for therapists’ competence, therapeutic alliance and patients’ symptoms.

Outcome	k	g	95%-CI	Q	I^2^
Main analysis					
Competence^1^	7	0.47	-0.14, 1.07	33.24***	79.1%
Alliance^2^	8	0.52	-0.24, 1.27	87.13***	within-study: 0% between-study: 88.98%
Patients’ symptoms^1^	9	-0.24	-0.48, 0.00	22.86**	65.71%
Sensitivity analysis (≥ 3 supervision sessions only)					
Competence^1^	4	0.74	0.20, 1.29	0.26	0%
Alliance^2^	5	0.92	-0.03, 1.87	20.20***	within-study: 0% between-study: 79.3%
Patients’ symptoms^1^	9	-0.24	-0.48, 0.00	22.86**	65.71%

k number of comparisons; ^1^two-level model; ^2^ three-level model; *p < .05, **p < 0.01, ***p < 0.001.

**Table 2:** Subgroup analyses for control condition.

Outcome	Subgroup	k	g	95%-CI	Q	I^2^	Meta-regression (p-value)
Competence	Passive	3	0.95	0.14, 1.76	16.36**	81.57%	0.097
	Active	4	0.12	-0.54, 0.78			
Alliance	Passive	4	1.12	0.21, 2.03	26.55***	62.42%	0.074
	Active	4	0.05	-0.76, 0.85			
Patients’ symptoms	Passive	6	-0.28	-0.54, 0.33	18.97**	57.75%	0.498
	Active	3	-0.11	-0.62, 0.07			

k number of comparisons; *p < .05, **p < 0.01, ***p < 0.001; assuming that all subgroups are sharing a common estimate of the between-study heterogeneity τ^2^.

Among the studies not included in the meta-analysis, Carmel et al. (38) reported a marginal significant effect of BITE Supervision compared to SAU on supervisees’ competence. Bearman et al. (35) also reported significantly higher competence in the Supervision+ (scaffolding and experiential learning) condition compared to SAU. Smith et al. (45) found significantly higher competence scores of live tele-conferencing supervision compared to no supervision and marginally significant higher competence of audio-tape supervision compared to no supervision. There was no difference between the two active supervision conditions. All uncontrolled studies predominantly reported higher competence at post-assessment, with the exception of Beidas et al. (36). The authors reported no significant effectof supervision quantity on the supervisee’s knowledge, but on their skills.

### Effects of supervision on therapeutic alliance

**Figure 3:**
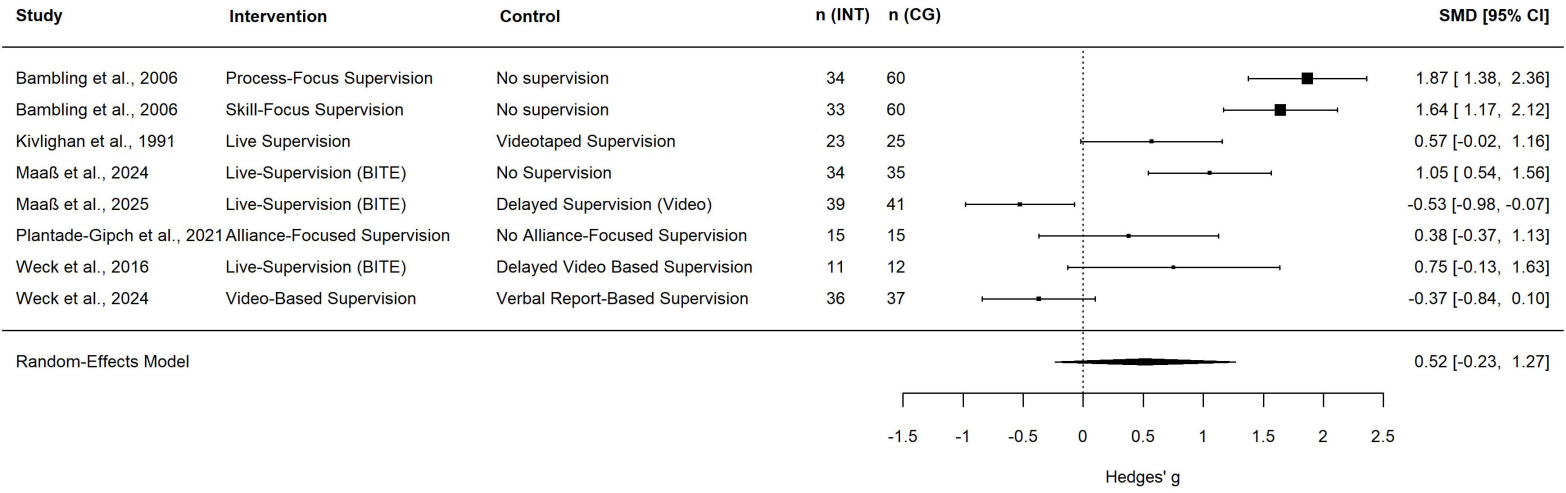
Forest plot of therapeutic alliance. Square size indicates study weight; a positive effect size indicates the superiority of the intervention group; n (INT), number of therapists in the intervention group; n (CG), number of therapists in the control group; SMD, standardized mean difference; CI, confidence interval; BITE, bug-in-the-eye supervision.

Eight studies reported effects of supervision on therapeutic alliance (13, 33, 40, 46–50). Seven trials could be included in the meta-analysis. Bambling et al. (33) included an intervention condition (Process-Focus Supervision) and an AC condition (Skill-Focus Supervision) which were compared to a passive No-Supervision control condition. The other trials had four active (see **Figure 3** for details) and two passive control conditions. A meta-analysis of these trials found a medium non-significant effect of the supervision intervention on therapeutic alliance (*g* = 0.52, 95%-CI [-0.24, 1.27], *k* = 8; see **Table 1** for the meta-analysis and **Figure 3** for the forest plot). Heterogeneity in the meta-analysis might not be important regarding within-study heterogeneity (*I^2^* = 0%) and substantial regarding between study heterogeneity (*I^2^* = 88.98%). When excluding trials with less than three supervision sessions (13, 40, 47) the effect size was large but also non-significant (*g* = 0.92, 95%-CI [-0.03, 1.87], *k* = 5; see **Table 1**). Heterogeneity remained substantial (*I^2^* = 79.3%). Compared to passive control conditions, supervision yielded a significant and large effect on the therapeutic alliance (*g* = 1.12, 95%-CI [0.21, 2.03], *k* = 4), while no effect was observed in comparison to ACs (*g* = 0.05, 95%-CI [-0.76, 0.85], *k* = 4). The subgroup analysis was non-significant (*p* = 0.074; see **Table 2**). The study that was not included in the meta-analysis reported that the quality of supervisory alliance was significantly related to the therapeutic alliance (50).

### Effects of supervision on patients’ symptoms

**Figure 4:**
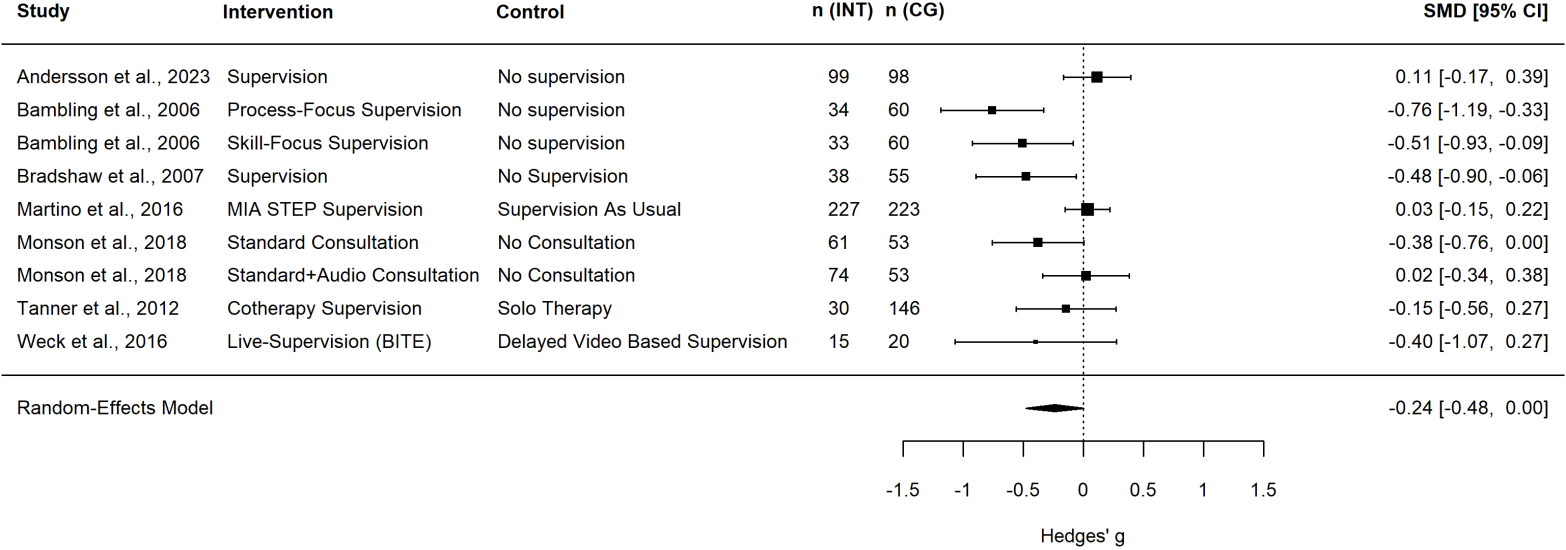
Forest plot of patients’ symptoms. Square size indicates study weight; a negative effect size indicates the superiority of the intervention group; n (INT), number of patients in the intervention group; n (CG), number of patients in the control group; SMD, standardized mean difference; CI, confidence interval; MIA STEP, Motivational Interviewing Assessment Supervisory Tools for Enhancing Proficiency; BITE, bug-in-the-eye supervision.

A total of nine studies reported effects of supervision on patients’ symptoms (33, 37, 39, 41, 46, 51–54). Of these, seven trials could be included in the meta-analysis. As described above, Bambling et al. (33) included an additional AC as well as Monson et al. (53), which included Standard and Audio Consultation as an intervention condition and Standard Consultation as an AC compared to a passive No-Supervision control condition. The other trials included two active control conditions (see **Figure 4**) and three passive control conditions. A meta-analysis of these trials found a small but non-significant effect of supervision on patients’ symptoms (*g* = -0.24, 95%-CI [-0.48, 0.00], *k* = 9; see **Table 1** for the meta-analysis and **Figure 4** for the forest plot). Heterogeneity in the meta-analysis was substantial (*I^2^* = 65.71%). There were no trials with less than three supervision sessions. In the subgroup analyses there was also a non-significant effect on patients’ symptoms when supervision is compared to passive controls (*g* = -0.28, 95%-CI [-0.28, 0.33], *k* = 6) or ACs (*g* = -0.11, 95%-CI [-0.62, 0.07], *k* = 6). Again, the subgroup analysis was non-significant (*p* = 0.498; see **Table 2**). Karlsruher (52) and Johnson et al. (39) also reported positive effects of supervision on patients’ symptoms.

### Effects of supervision on therapists’ adherence and fidelity

Seven studies reported the effects of supervision on therapists’ adherence (33, 36, 41, 45, 55–57). One study also reported therapists’ fidelity, summarizing competence, differentiation and adherence in one outcome (35). Martino et al. (41) as well as Dorsey et al. (56) reported no significant difference between active supervision conditions regarding therapist’s adherence in their controlled trials. Dorsey et al. (56) additionally compared the two conditions Symptom and fidelity monitoring (SFM) and SFM + Behavioral Rehearsal (BR) with SAU (Phase I as reported in 57) and also found no significant difference regarding therapist’s adherence. Smith et al. (45) reported significantly higher adherence scores of Tele-Conferencing (live) Supervision compared to no supervision and marginally significant higher adherence of Tape Supervision compared to no supervision. There was no difference between the two active supervision conditions. Bambling et al. (33) found no correlation between adherence and symptom reduction nor therapeutic alliance. In contrast, Bearman et al. (35) found that the supervision intervention had a significant impact on therapists’ fidelity. In the uncontrolled studies, positive effects of supervision on adherence were reported: Meza et al. (57) evaluated adherence depending on supervision style in a specific session and found that clinicians receiving supportive-directive supervision were more likely to deliver adherent to the manual than clinicians receiving supportive supervision. Beidas et al. (36) reported that supervision hours significantly predicted adherence and Anderson et al. (55) also reported significant improvement in therapist’s adherence after the supervision intervention.

### Subgroup analyses and publication bias

Since we could only include a small number of studies per outcome, we were unable to assess publication bias (30). Due to the small number of included studies we did not conduct additional moderator analyses and are unable to statistically address the research question about possible moderators.

### Certainty of evidence (GRADE)

The certainty of evidence was rated very low for all outcomes supervisees' competence, therapeutic alliance and patients' symptoms. due to serious risk of bias, serious inconsistency and serious imprecision. The detailed GRADE Evidence Profile can be found in the **Appendix G**.

## Discussion

To the best of our knowledge, this is the first comprehensive systematic review to include a meta-analysis on the effectiveness of clinical supervision. The review included a total of 31 controlled and uncontrolled studies examining the effectiveness of supervision, while the meta-analyses included seven studies each for therapists’ competence, therapeutic alliance, and for patients’ symptoms. Across these studies, a variety of supervision interventions were utilized for diverse populations. Supervision interventions varied regarding the supervision model and included BITE supervision, supervision with video or audio recordings or verbal report supervisions. Also, they sometimes focused on learning a specific manual (e.g. CPT, DBT, …) or method (e.g. motivational interviewing). These interventions are compared to various control conditions ranging from passive controls, SAU to mostly only marginally different active controls. The outcomes reported were heterogeneous, with a maximum of 15 studies reporting the same outcome (therapists’ competence). In general, the efficacy of supervision has been demonstrated in a variety of study designs with respect to a range of outcomes, while heterogeneity regarding the results for all outcomes but also regarding the operationalization and the assessment was high.

The meta-analysis for therapists’ competence yielded a medium but non-significant effect and substantial heterogeneity in the effect size. Regarding therapeutic alliance, we found a medium but non-significant effect in the meta-analysis. It should be noted that in both cases there was substantial heterogeneity, consequently the certainty of evidence was low. When excluding studies with interventions with less than three supervision sessions, the effect sizes tended to be higher and even significant for therapeutic alliance. Looking at effect sizes for therapists’ competence on a study level, large effect sizes for passive control conditions were observed while effect sizes got smaller and non-significant for active controls. Also, for therapeutic alliance when the supervision intervention was compared with a passive control condition, the effect sizes were consistently significant, yet they were non-significant when the intervention was compared with an active control. This observation is also supported by the explorative subgroup analysis which yielded large effects for both outcomes when the intervention was compared to a passive control condition. Yet, due to the small number of studies included these observations should be interpreted with caution, as subgroup analyses usually are recommended for computation if the meta-analysis for an outcome contains at least *k =* 10 studies or at least k = 4 studies per subgroup (27, 28). When the results of the meta-analysis are considered in relation to the studies that were not included in the quantitative analysis (i.e. controlled studies where data could not be obtained and uncontrolled studies), it appears that supervision, in general, seems effective in promoting therapists’ competence and therapeutic alliance while there may be no superiority of a specific supervision model. This would be consistent with the observations from previous reviews (8, 17). However, further quantitative research is needed to support this hypothesis. It should also be noted that the studies which produced negative effect sizes in the meta-analyses for therapists’ competence and therapeutic alliance both were short supervision interventions of only one or two supervision sessions with university students and an active supervision intervention as control condition (40, 47).

Regarding patients’ symptoms we found a small but non-significant effect in the meta-analysis, but heterogeneity was large. Studies included patients with various disorders (e.g. depression, substance use disorder, PTSD, mixed) and also various different, not always standardized, outcome measures (e.g. BDI, 58; Substance Use Calendars). This clinical and methodological variability may contribute to the heterogeneity of findings. Furthermore, variations in the efficacy of psychotherapy and specific psychotherapeutic rationales across different disorders might undermine the effects of supervision in cases where the psychotherapeutic intervention produces only small or inconsistent effects. For example, the effect of psychotherapy for depression is small, while the effect of psychotherapy for PTSD is medium (59). Again, effect sizes were predominantly higher for passive comparison conditions, which was also indicated by the subgroup analyses. Andersson et al. (51) found a positive effect size even though comparing the supervision intervention with a passive comparison. This might be explained with the randomization process in this particular trial: Andersson et al. (51) randomized the patients discussed in supervision, not the supervisees who received supervision. As the authors also discuss, the supervisees that received supervision for another case might benefit from these consultations and transfer their learnings to the other cases. Monson et al. (53) reported an effect size close to zero when comparing Standard + Audio Consultation with no supervision. The authors suggest that this might be due to the reduced time available for discussion of the cases because auf listening to the audio sequences during supervision sessions. Martino et al. (41) also reported a small positive effect (i.e. negative effect size) of a motivational interviewing specific supervision compared to SAU on patients’ symptoms. The reason for this result could be that the measurement of the outcome, which was a Substance Use Calendar (self-reported substance use). In general, this effect indicates that patients tend to benefit from the supervision of their therapists. However, the results of this analysis should be interpreted with caution, as the effect is only small and only a few studies were included in the analysis, and the effect was non-significant.

The precise mechanisms through which supervision exerts a positive effect on patients remain unclear. Some studies report therapeutic alliance to be a predictor of better treatment outcomes and less dropout (60, 61). Yet, in a meta-analysis Webb et al. (62) found that neither adherence nor competence were related to patient outcomes in their meta-analysis and pooled effects were close to zero. In a small meta-analysis Zarafonitis et al. (63) reported a small but significant correlation of therapists’ competence on patients’ symptom reduction but no correlation for therapists’ adherence and patients’ symptom reduction. However, the analysis only included seven trials for each, competence and adherence. In contrast, Keum and Wang (10) as well as Park et al. (15) found evidence of process supervision variables being positively correlated with therapeutic process variables. In order to explain a potential mediating role of outcomes such as therapists’ competence, adherence and therapeutic alliance on patients’ symptom reduction through meta-analysis more studies reporting both, outcomes on the therapist/therapy-level as well as patients’ symptoms, are needed.

There is a conceptual overlap among these constructs assessed in the included studies, such as competence, alliance, and patient outcomes. These variables are theoretically interconnected and may influence one another. This probable mediating effect could not be studied in the analysis.

The substantial heterogeneity in effect sizes for all outcomes indicates that there may be moderators of the supervision effects. The exploratory subgroup analysis suggests that some heterogeneity can be explained by the type of control group (active vs. passive). However, even after the subgroup analyses heterogeneity remains high, and more studies are needed to explain heterogeneity using meta-regression. For example, the therapists’ level of experience, the number of supervision sessions, and their duration may also contribute to heterogeneity. However, these moderators were not accounted for and should be addressed in future research. In general, our results support the suggestions by Alfonsson et al. (8) and White (64) that there is a clear need for more high-quality research on the effects of supervision, especially with a control group that does not only focus on technical issues (such as video-based supervision vs. BITE supervision) but allows to inform on model specific aspects.

It should also be noted that this review only included studies that explicitly examined supervision interventions. There are other survey studies that provide comprehensive insight into the effects of supervision in psychotherapeutic care. One example of this is the study by Forshammar Geisler et al. 65, which demonstrated that therapists who regularly undergo supervision exhibit fewer symptoms of work exhaustion.

Studies with an AC often addressed more technical questions, e.g. comparing BITE supervision with delayed video-based supervision or video-based supervision with verbal report-based supervision (e.g. 40, 46–48). Studies comparing conceptually different supervision models (i.e. process-oriented vs. client-centered, vs. self-awareness-oriented vs. skills-oriented etc.; e.g. 33) are mostly still lacking. More studies with different supervision models – not only methods – are needed to understand what makes supervision effective.

### Limitations

The results of this first meta-analysis on supervision in the context of psychotherapy must be interpreted with caution. A key limitation of the present study is the extensive inclusion criteria for the studies. In order to provide a general overview of the effectiveness of supervision interventions in combination with the small number of included studies in previous reviews, it was necessary to set broad inclusion criteria. Consequently, the populations, interventions and study designs are heterogeneous. Another limitation of this study is the heterogeneity of the approaches adopted for the analysis of the effects of the supervision intervention. The standardization of the interventions varied considerably across the included studies. This further results in increased heterogeneity in effect sizes and wider confidence intervals. If data needed for the effect size calculation were not described in the article, study authors were contacted. As we were unable to obtain some data, this might result in selection bias. Regarding the outcomes, it should be noted that their measurement varied across studies, since the inclusion criteria allowed self-report measures and independent ratings. The instruments used were not always standardized, with some studies utilizing self-developed scales or tests. In addition, competence, skills and knowledge were summarized in the outcome supervisees’ competence. All this results in further heterogeneity in the effect sizes. Additionally, the quality of the studies was predominantly rated as low. Consequently, the GRADE quality of evidence for all outcomes was very low. For these reasons, the results of the meta-analysis presented here should be interpreted with caution and in the context with the general overview of included studies of the review.

### Implications and conclusions

This is the first and comprehensive review and meta-analysis on supervision. We used a search strategy yielding over 15,000 titles and abstracts. This work represents a promising first step in the quantification of the effects of supervision through meta-analysis, though substantial work and further research remain necessary.

In general, supervision tends to show a small to medium positive effect on both the supervisee and the patient, although the exact mechanisms of action are to be investigated. Yet, these effects are tentative and require confirmation by higher-quality studies. While the findings of our subgroup analysis are comparable to effects in treatment-focused research this itself is informative, as it underlines that supervision might reach a plateau of effectiveness once certain basic characteristics are met. However, further investigation is needed to learn what these are.

Future studies should also investigate possible moderators to explain the substantial heterogeneity in effect sizes found in this analysis. Therefore, there is a need for further controlled trials of different supervision interventions, especially with an active control condition in the form of SAU, and passive controls, as well as supervision models as stated above, are needed. Such additional research would facilitate a more comprehensive understanding of the mechanisms of supervision and the development of criteria for effective supervision that are applicable in clinical settings. This will also facilitate further investigation of the above assumption that supervision is effective, while clarifying whether there is no superiority of a specific supervision model, or whether this observation is merely due to comparator groups focusing on technical details rather than conceptual distinctions between supervision models. Subsequent research should also aim to identify effective supervision characteristics that are feasible and not overly cost-intensive, as the latter has been discussed as a limitation in various studies.

Future studies on the impact of supervision on patients’ symptoms should focus on the treatment of disorders for which therapy has been shown to have large effects in reducing symptoms. Otherwise, the effect of supervision may be undermined by the limited efficacy of the underlying therapeutic intervention.

Additionally, it would be of interest to examine therapist- and therapy-related outcomes as a mediator of the effect of the supervision intervention on the patients’ symptoms. To facilitate this, it is essential that a consensus can be reached among supervision studies on the assessment and reporting of primary outcomes. More data on the characteristics of supervisees and supervisors should be reported in order to account for factors such as age, experience and theoretical orientation, as well as supervisors’ qualifications in the analysis. By achieving this, individual studies can be made more comparable and more suitable for inclusion in an overall effect in meta-analyses and outcomes would be eligible for mediator meta-analyses. Moreover, other outcomes that would be of interest were hardly reported, such therapists’ readiness to treat and the duration of therapy.

In conclusion the results indicate that supervision may have positive effects on the supervisee and their patients even though the currently available research on supervision is heterogenous. Yet, despite the wide range of available training programs for supervisors, many of which claim to teach effective supervisory techniques, there is still limited empirical evidence on which specific components make supervision effective. This highlights the need for further research to inform evidence-based training and practice.

## Data Availability

The raw data supporting the conclusions of this article will be made available by the authors, without undue reservation.
