# Correction: The effects of clinical supervision on supervisees and patient outcomes in psychotherapy – a systematic review and meta-analysis

**DOI:** 10.3389/fpsyt.2025.1764015

**Published:** 2026-01-21

**Authors:** Bianca Schreyer, Cosima Leithner, Rebekka Eilers, Katharina Gossmann, Rita Rosner

**Affiliations:** 1 Department of Psychology, Faculty of Philosophy and Education, Catholic University Eichstaett-Ingolstadt, Eichstätt, Germany; 2 Department of Psychology, Faculty of Psychology and Educational Sciences, Ludwig-Maximilians-University Munich, Munich, Germany

**Keywords:** supervision, psychotherapy, therapists in training, therapist competence, therapeutic alliance, patient symptoms, meta-analysis

The reference for Milne and Watkins (2014) was erroneously written as “Milne DL, Watkins CE. (2014)”. It should be:

“Milne DL, Watkins CE. “Defining and Understanding Clinical Supervision”. In: Watkins CE, Milne DL, editors. *The Wiley International Handbook of Clinical Supervision.* Wiley (2014). pp. 1–19”.

In the **Introduction**, the names “Maaß et al.” were missing in front of the citation (17).

A correction has been made to the section **Introduction**, in the first sentence of the fourth paragraph:

“Vezer (16) and Maaß et al. (17) both conducted more recent reviews on the literature focusing on live supervision as a specific supervision technique.”

The original version of this article has been updated.

