# Supplementary material for: The effects of clinical supervision on supervisees and patient outcomes in psychotherapy – a systematic review and meta-analysis
Source: Front Psychiatry. 2025 Nov 21;16:1705578. doi: 10.3389/fpsyt.2025.1705578 (PMC12679832; doi:10.3389/fpsyt.2025.1705578)
Supplement: Supplementary file 1 [file DataSheet1.docx]

**Appendix**

[Appendix A: Search strategy 2](#_Toc212019299)

[Appendix B: Full citations of included studies 4](#_Toc212019300)

[Appendix C: Potentially relevant studies that were read in full-text form but excluded from the review 7](#_Toc212019301)

[Appendix D Overview of included studies 20](#_Toc212019302)

[Appendix E: Main outcomes of included studies 30](#_Toc212019303)

[Appendix F: Risk of Bias Rating 35](#_Toc212019304)

[Appendix G: GRADE Evidence Profile 37](#_Toc212019305)

# Appendix A: Search strategy

**Pubmed - 5,734 results**

(“Psychiatry”[mh] OR “Psychotherapy”[mh] OR ”psychiatr*”[tiab] OR “psychotherap*”[tiab] OR (“social work*”[tiab] AND (“psychotherap*”[tiab] OR (“psycho*”[tiab] AND “therap*”[tiab]))) OR “mental health nurs*”[tiab] OR ((“psycho*”[tiab] OR “systemic”[tiab] OR ”psychoanalytic”[tiab] OR ”psychodynamic”[tiab] OR ”cognitive”[tiab] OR ”behavioral”[tiab]) AND (“therap*”[tiab] OR “intervention*”[tiab] OR “counsel*”[tiab] OR “treatment*”[tiab])))

AND

(“case consultation*”[tiab] OR “supervisor*”[tiab] OR “supervisee*”[tiab] OR ((“clinical”[tiab] or “professional”[tiab] or “competency based”[tiab] or “bug in the eye”[tiab] or “bug in the ear”[tiab] or “BITE”[tiab] or “live”[tiab] or “practicum”[tiab] or “therapeutic”[tiab]) AND “supervis*”))

AND (humans[Filter])

**PsycINFO via EBSCO - 4,522 results**

(MA “Psychiatry” OR MA “Psychotherapy” OR TI ”psychiatr*” OR TI “psychotherap*” OR (TI “social work*” AND (TI“psychotherap*” OR (TI “psycho*” AND TI “therap*”))) OR TI “mental health nurs*” OR AB ”psychiatr*” OR AB “psychotherap*” OR (AB “social work*” AND (AB “psychotherap*” OR (AB “psycho*” AND AB “therap*”))) OR AB “mental health nurs*” OR ((TI “psycho*” OR TI “systemic” OR TI ”psychoanalytic” OR TI ”psychodynamic” OR TI ”cognitive” OR TI ”behavioral”) AND (TI “therap*” OR TI “intervention*” OR TI “counsel*” OR TI “treatment*”)) OR ((AB “psycho*” OR AB “systemic” OR AB ”psychoanalytic” OR AB ”psychodynamic” OR AB ”cognitive” OR AB ”behavioral”) AND (AB “therap*” OR AB “intervention*” OR AB “counsel*” OR AB “treatment*”)))

AND

(KW “supervis*” OR MA “professional supervision” OR MA “practicum supervision” OR TI “supervisor*” OR TI ”supervisee*” OR TI ”case consultation*” OR AB “supervisor*” OR AB ”supervisee*” OR AB ”case consultation*” OR ("clinical" OR "professional" OR "competency based" OR "bug in the eye" OR "bug in the ear" OR "BITE" OR "live" OR "practicum" OR "therapeutic") NEAR/3 "supervis*")

**Web of Science - 4,700 results**

TS=(“psychiatr*" OR "psychotherap*" OR ("social work*" AND ("psychotherap*" OR ("psycho*" AND "therap*"))) OR "mental health nurs*" OR (("psycho*" OR "systemic" OR "psychoanalytic" OR "psychodynamic" OR "cognitive" OR "behavioral") AND ("therap*" OR "intervention*" OR "counsel*" OR "treatment*")))

AND

(AK=(supervision) OR TS=("professional supervision" OR "practicum supervision" OR "case consultation*" OR "supervisor*" OR "supervisee*" OR ("clinical" OR "professional" OR "competency based" OR "bug in the eye" OR "bug in the ear" OR "BITE" OR "live" OR "practicum" OR "therapeutic") NEAR/3 "supervis*"))

**Epistemonikos – 11 results**

(”psychiatr*” OR “psychotherap*” (“social work*” AND (“psychotherap*” OR (“psycho*” AND “therap*”))) OR “mental health nurs*” OR ((“psycho*” OR “cognitive behavioral” OR “systemic” OR ”psychoanalytic” OR ”psychodynamic” OR ”cognitive” OR ”behavioral”) AND (“therap*” OR “intervention*” OR “counsel*” OR “treatment*”)))

AND

("professional supervision" OR "practicum supervision" OR "case consultation*" OR "supervisor*" OR "supervisee*" OR ("clinical" OR "professional" OR "competency based" OR "bug in the eye" OR "bug in the ear" OR "BITE" OR "live" OR "practicum" OR "therapeutic") AND "supervis*")

+ filter primary studies

**Cochrane Library - 2,251 results**

(psychiatr* OR psychotherap* (social work* AND (psychotherap* OR (psycho* AND therap*))) OR mental health nurs* OR ((psycho* OR systemic OR psychoanalytic OR psychodynamic OR cognitive OR behavioral) AND (therap* OR intervention* OR counsel* OR treatment*)))

AND

(case consultation* OR supervisor* OR supervisee* OR “clinical supervision” OR “practicum supervision” OR “professional supervision” OR “competency based supervision” OR bug in the eye OR bug in the ear)

**EMBASE via OVID - 3,214 results**

(psychiatry/ or psychotherapy/ or (psychiatr*.ti,ab. or psychotherap*.ti,ab.) or (social work*.ti,ab. and (psychotherap*.ti,ab. or (psycho*.ti,ab. and therap*.ti,ab.))) or mental health nurs*.ti,ab. or ((psycho*.ti,ab. or systemic.ti,ab. or psychoanalytic.ti,ab. or psychodynamic.ti,ab. or cognitive.ti,ab. or behavioral.ti,ab.) and (therap*.ti,ab. or intervention*.ti,ab. or counsel*.ti,ab. or treatment*.ti,ab.)))

and (professional supervision/ or practicum supervision/ or case consultation*.ti,ab. or supervisor*.ti,ab. or supervisee*.ti,ab. or ((clinical or professional or competency based or bug in the eye or bug in the ear or BITE or live or practicum or therapeutic) adj3 supervis*))

# Appendix B: Full citations of included studies

Alfonsson, S., Lundgren, T., & Andersson, G. (2020). Clinical supervision in cognitive behavior therapy improves therapists’ competence: A single-case experimental pilot study. *Cognitive Behaviour Therapy*, *49*(5), 425–438. <https://doi.org/10.1080/16506073.2020.1737571>

Anderson, T., Crowley, M. E. J., Patterson, C. L., & Heckman, B. D. (2012). The influence of supervision on manual adherence and therapeutic processes. *Journal of Clinical Psychology*, *68*(9), 972–988. <https://doi.org/10.1002/jclp.21879>

Andersson, G., Käll, A., Juhlin, S., Wahlström, C., Fine Licht, E. de, Färdeman, S., Franck, A., Tholcke, A., Nachtweij, K., Fransson, E., Vernmark, K., Ludvigsson, M., & Berg, M. (2023). Free choice of treatment content, support on demand and supervision in internet-delivered CBT for adults with depression: A randomized factorial design trial. *Behaviour Research and Therapy*, *162*, 1–11. <https://doi.org/10.1016/j.brat.2023.104265>

Bambling, M., King, R., Raue, P., Schweitzer, R., & Lambert, W. (2006). Clinical supervision: its influence on client-rated working alliance and client symptom reduction in the brief treatment of major depression, *16*(3), 317–331. <https://doi.org/10.1080/10503300500268524>

Bartle-Haring, S., Silverthorn, B. C., Meyer, K., & Toviessi, P. (2009). Does live supervision make a difference? A multilevel analysis. *Journal of Marital and Family Therapy*, *35*(4), 406–414. <https://doi.org/10.1111/j.1752-0606.2009.00124.x>

Bearman, S. K., Schneiderman, R. L., & Zoloth, E. (2017). Building an evidence base for effective supervision practices: An analogue experiment of supervision to increase EBT fidelity. *ADMINISTRATION and POLICY in MENTAL HEALTH and MENTAL HEALTH SERVICES RESEARCH*, *44*(2), 293–307. <https://doi.org/10.1007/s10488-016-0723-8>

Beidas, R. S., Edmunds, J. M., Marcus, S. C., & Kendall, P. C. (2012). Training and consultation to promote implementation of an empirically supported treatment: A randomized trial. *Psychiatric Services*, *63*(7), 660–665. <https://doi.org/10.1176/appi.ps.201100401>

Bradshaw, T., Butterworth, A., & Mairs, H. (2007). Does structured clinical supervision during psychosocial intervention education enhance outcome for mental health nurses and the service users they work with? *Journal of Psychiatric and Mental Health Nursing*, *14*(1), 4–12. <https://doi.org/10.1111/j.1365-2850.2007.01021.x>

Carmel, A., Villatte, J. L., Zachary Rosenthal, M., Chalker, S., & Comtois, K. A. (2016). Applying Technological Approaches to Clinical Supervision in Dialectical Behavior Therapy: A Randomized Feasibility Trial of the Bug-in-the-Eye (BITE) Model. *Cognitive and Behavioral Practice*, *23*(2), 221–229. <https://doi.org/10.1016/j.cbpra.2015.08.001>

Dorsey, S., AlRasheed, R [Rashed], Kerns, S. E., Meza, R. D., Triplett, N., Deblinger, E., Jungbluth, N., Berliner, L., Naithani, L., & Pullmann, M. D. (2025). A randomized controlled trial testing supervision strategies in community mental health. *Implementation Research and Practice*, *6*, 26334895251330523. <https://doi.org/10.1177/26334895251330523>

Ellis, M. V., & Ladany, N. (1997). Inferences concerning supervisees and clients in clinical supervision: An integrative review. In C. E. Watkins (Ed.), *Handbook of psychotherapy supervision* (pp. 447–507). John Wiley & Sons, Inc.

Johnson, L. L., Phythian, K., Wong, B., McGill, C., Hinds, A., Choate, E., Zelcer, H., Mendez, E., Movahedi, Y., & Friedberg, R. D. (2023). Training Clinical Staff in Genuine CBT: One Large Agency’s Preliminary Story. *International Journal of Cognitive Therapy*, *16*(4), 479–496. <https://doi.org/10.1007/s41811-023-00179-9>

Karlsruher, A. E. (1976). The influence of supervision and facilitative conditions on the psychotherapeutic effectiveness of nonprofessional and professional therapists. *American Journal of Community Psychology*, *4*(2), 145–154. <https://doi.org/10.1007/BF00881936>

Kivlighan, D. M., Angelone, E. O., & Swafford, K. G. (1991). Live supervision in individual psychotherapy: Effects on therapist’s intention use and client’s evaluation of session effect and working alliance. *Professional Psychology: Research and Practice*, *22*(6), 489–495. <https://doi.org/10.1037/0735-7028.22.6.489>

Krall, H. (2022). Supervision von Psychotherapeut*innen in Ausbildung – Welchen Mehrwert bringen psychodramatische und soziometrische Verfahren? *Zeitschrift Für Psychodrama Und Soziometrie*, *21*(S2), 135–151. <https://doi.org/10.1007/s11620-022-00697-2>

Maaß, U., Eisert, K., Ghalib, J., Kühne, F., & Weck, F. (2025). Live versus delayed supervision: A randomized controlled trial with psychology students. *Psychotherapy (Chicago, Ill.).* Advance online publication. <https://doi.org/10.1037/pst0000572>

Maaß, U., Kühne, F., Ay-Bryson, D. S., Heinze, P. E., & Weck, F. (2024). Efficacy of live-supervision regarding skills, anxiety and self-efficacy: a randomized controlled trial. *The Clinical Supervisor*, *43*(1), 1–21. <https://doi.org/10.1080/07325223.2023.2267528>

Martino, S., Paris, M., Añez, L., Nich, C., Canning-Ball, M., Hunkele, K., Olmstead, T. A., & Carroll, K. M. (2016). The Effectiveness and Cost of Clinical Supervision for Motivational Interviewing: A Randomized Controlled Trial. *Journal of Substance Abuse Treatment*, *68*, 11–23. <https://doi.org/10.1016/j.jsat.2016.04.005>

Meza, R. D., AlRasheed, R., Pullmann, M. D., & Dorsey, S. (2023). Clinical supervision approach predicts evidence-based trauma treatment delivery in children’s mental health, *13*, 1072844. <https://doi.org/10.3389/fpsyt.2022.1072844>

Monson, C. M., Shields, N., Suvak, M. K., Lane, J. E. M., Shnaider, P., Landy, M. S. H., Wagner, A. C., Sijercic, I., Masina, T., Wanklyn, S. G., & Stirman, S. W. (2018). A randomized controlled effectiveness trial of training strategies in cognitive processing therapy for posttraumatic stress disorder: Impact on patient outcomes. *Behaviour Research and Therapy*, *110*, 31–40. <https://doi.org/10.1016/j.brat.2018.08.007>

Ng, R. M. K., & Cheung, M. S. M. (2007). Supervision of cognitive behavioural therapy for psychosis: A Hong Kong experience. *Hong Kong Journal of Psychiatry*, *17*(4).

Ögren, M.‑L., & Jonsson, C.‑O. (2004). Psychotherapeutic Skill Following Group Supervision According to Supervisees and Supervisors. *The Clinical Supervisor*, *22*(1), 35–58. <https://doi.org/10.1300/J001v22n01_04>

Plantade-Gipch, A., Blanchet, A., & Drouin, M.‑S. (2021). Can alliance-focused supervision help improve emotional involvement and collaboration between client and therapist? *European Journal of Psychotherapy & Counselling*, *23*(1), 26–42. <https://doi.org/10.1080/13642537.2021.1881138>

Rakovshik, S. G., McManus, F., Vazquez-Montes, M., Muse, K., & Ougrin, D. (2016). Is supervision necessary? Examining the effects of internet-based CBT training with and without supervision. *Journal of Consulting and Clinical Psychology*, *84*(3), 191–199. <https://doi.org/10.1037/ccp0000079>

Reese, R. J., Aldarondo, F., Anderson, C. R., Lee, S.‑J., Miller, T. W., & Burton, D. (2009). Telehealth in clinical supervision: A comparison of supervision formats. *Journal of Telemedicine and Telecare*, *15*(7), 356–361. <https://doi.org/10.1258/jtt.2009.090401>

Reese, R. J., Usher, E. L., Bowman, D. C., Norsworthy, L. A., Halstead, J. L., Rowlands, S. R., & Chisholm, R. R. (2009). Using client feedback in psychotherapy training: An analysis of its influence on supervision and counselor self-efficacy. *Training and Education in Professional Psychology*, *3*(3), 157–168. <https://doi.org/10.1037/a0015673>

Smith, J. L., Carpenter, K. M., Amrhein, P. C., Brooks, A. C., Levin, D., Schreiber, E. A., Travaglini, L. A., Hu, M.‑C., & Nunes, E. V. (2012). Training substance abuse clinicians in motivational interviewing using live supervision via teleconferencing. *Journal of Consulting and Clinical Psychology*, *80*(3), 450–464. <https://doi.org/10.1037/a0028176>

Tanner, M. A., Gray, J. J., & Haaga, D. A. F. (2012). Association of cotherapy supervision with client outcomes, attrition, and trainee effectiveness in a psychotherapy training clinic. *Journal of Clinical Psychology*, *68*(12), 1241–1252. <https://doi.org/10.1002/jclp.21902>

Weck, F., Jakob, M., Neng, J. M. B., Höfling, V., Grikscheit, F., & Bohus, M. (2016). The Effects of Bug-in-the-Eye Supervision on Therapeutic Alliance and Therapist Competence in Cognitive-Behavioural Therapy: A Randomized Controlled Trial. *Clinical Psychology & Psychotherapy*, *23*(5), 386–396. <https://doi.org/10.1002/cpp.1968>

Weck, F., Maaß, U., Paunov, T., Heinze, P. E., & Kühne, F. (2024). Clinical supervision based on video vs. Verbal report: A randomized controlled trial. *Cognitive Behaviour Therapy*, 1–19. <https://doi.org/10.1080/16506073.2024.2434016>

Wittenborn, A. K., Subramaniam, S., Morgan, P. C., & Tseng, C.‑F. (2022). Effects of adding video feedback to emotionally focused therapy supervision: A concurrent multiple-baseline across subjects design. *Journal of Marital and Family Therapy*, *48*(4), 1059–1074. <https://doi.org/10.1111/jmft.12586>

# Appendix C: Potentially relevant studies that were read in full-text form but excluded from the review

| Reference | Reason for Exclusion |
| --- | --- |
| Abramowitz, S. I., Weitz, L. J., & James, C. R. (1974). Supervisor self-concept and self-concept deterioration among psychotherapy trainees. *Journal of Clinical Psychology*, *30*(3), 300–302. | Wrong intervention |
| Abrar, S., Hafeez, A., Khan, M. N., Haroon, M. Z., & Khan, A. (2021). Effectiveness of DIMCI training in district Abbottabad, Khyber Pakhtunkhwa: An external pilot study. *Journal of Ayub Medical College, Abbottabad: JAMC*, *33*(4), 602–606. | Wrong study design |
| Accurso, E. C., Taylor, R. M., & Garland, A. F. (2011). Evidence-based practices addressed in community-based children's mental health clinical supervision. *Training and Education in Professional Psychology*, *5*(2), 88–96. https://doi.org/10.1037/a0023537 | No supervisee or patient related outcomes |
| Adams, L. J., MacLean, R. R., Portnoy, G. A., Beauvais, J., & Stacy, M. A. (2023). Psychology trainee and supervisor perspectives of multicultural supervision. *Psychological Services*, *20*(Suppl 1), 78–85. https://doi.org/10.1037/ser0000643 | Wrong study design |
| Addleton, R. L., Tratnack, S. A., & Donat, D. C. (1991). Hospital-based multidisciplinary training in the care of seriously mentally ill patients. *Hospital & Community Psychiatry*, *42*(1), 60–61. https://doi.org/10.1176/ps.42.1.60 | Wrong study design |
| Aldahadha, B., & Karaki, W. (2022). Effectiveness of two clinical peer supervision models on practising practicum students. *Counselling and Psychotherapy Research*, *22*(4), 1068–1076. https://doi.org/10.1002/capr.12491 | Wrong participant characteristics |
| Allen, K. W., Tolchard, B., & Battersby, M. (2000). Behavioural psychotherapy training for nurses in Australia: A pilot program. Australian and New Zealand Journal of Mental Health Nursing, 9(2), 75–81. https://doi.org/10.1046/j.1440-0979.2000.00161.x | Wrong study design |
| Alpern A.N., McLean K.A., Marciel K.K., Zhang J., Riekert K.A. & Quittner A.L. (2012), Poster Session Abstracts. Pediatr. Pulmonol., 47: 223-446. https://doi.org/10.1002/ppul.22682 | Not peer-reviewed |
| Alpern, A. N., McLean, K. A., Becker, E. M., Riekert, K. A., & Quittner, A. L. (2016) Poster session abstracts. Pediatric Pulmonology, 51(S45), S194–S485. https://doi.org/10.1002/ppul.23576 | Not peer-reviewed |
| Fernández-Álvarez, H., & Del Grazioso, M. P. (2023). Desafíos de la Supervisión en Psicoterapia. *Revista De Psicoterapia*, *34*(126), 1–8. https://doi.org/10.5944/rdp.v34i126.38692 | Language not english or german |
| An, H. Y., Goodyear, R. K., Seo, Y. S., Garrison, Y., Baek, K. Y., & Cho, H. J. (2020). Supervisor style as a predictor of counseling supervision relationship quality and supervisee satisfaction: perceptions of U.S. and South Korean supervisees. *Asia Pacific Education Review*, *21*(3), 487–504. https://doi.org/10.1007/s12564-020-09637-x | Wrong study design |
| Anderson, T., Crowley, M. J., Binder, J. L., Heckman, B. D., & Patterson, C. L. (2017). Does the supervisor's teaching style influence the supervisee's learning prescribed techniques? *Psychotherapy Research: Journal of the Society for Psychotherapy Research*, *27*(5), 549–557. https://doi.org/10.1080/10503307.2015.1136442 | Wrong study design |
| Arczynski, A. V., & Morrow, S. L. (2017). The complexities of power in feminist multicultural psychotherapy supervision. *Journal of Counseling Psychology*, *64*(2), 192–205. https://doi.org/10.1037/cou0000179 | Wrong study design |
| Årling, M. (1998). Supervision in Psychotherapy: An educational experiment in integrative metalearning. *Transactional Analysis Journal*, *28*(3), 224–233. https://doi.org/10.1177/036215379802800306 | Wrong intervention |
| Arnott, S., Dorkins, E., & Aylard, P. (1996). “The Sorcerer and the Apprentice”. *Psychiatric Bulletin*, *20*(10), 609–611. https://doi.org/10.1192/pb.20.10.609 | Wrong study design |
| Ashmore, R., & Carver, N. (2000). Clinical supervision in mental health nursing courses. *British Journal of Nursing (Mark Allen Publishing)*, *9*(3), 171–176. https://doi.org/10.12968/bjon.2000.9.3.171 | Wrong study design |
| Ashmore, R., Carver, N., Clibbens, N., & Sheldon, J. (2012). Lecturers' accounts of facilitating clinical supervision groups within a pre-registration mental health nursing curriculum. *Nurse Education Today*, *32*(3), 224–228. https://doi.org/10.1016/j.nedt.2011.04.010 | Wrong study design |
| Authier, J., & Gustafson, K. (1975). Application of supervised and nonsupervised microcounseling paradigms in the training of paraprofessionals. *Journal of Counseling Psychology*, *22*(1), 74–78. https://doi.org/10.1037/h0076140 | Wrong intervention |
| Authier, J., & Gustafson, K. (1976). Application of supervised and nonsupervised microcounseling paradigms in the training of registered and licensed practical nurses. *Journal of Consulting and Clinical Psychology*, *44*(5), 704–709. https://doi.org/10.1037/0022-006X.44.5.704 | Wrong intervention |
| Axelsson, S., Kihlberg, S., Davis, P., & Nyström, M. B. T. (2024). Psychotherapy students' experiences of supervisee‐centred supervision based on deliberate practice, feedback‐informed treatment and self‐compassion. *Counselling and Psychotherapy Research*, *24*(2), 719–733. https://doi.org/10.1002/capr.12708 | Wrong study design |
| Bailin, A., & Bearman, S. K. (2021). Coverage of EBT practices in routine clinical supervision for youth: how much overlap with the evidence base? *The Clinical Supervisor*, *40*(1), 29–48. https://doi.org/10.1080/07325223.2020.1844107 | Wrong study design |
| Bailin, A., Bearman, S. K., & Sale, R. (2018). Clinical Supervision of Mental Health Professionals Serving Youth: Format and Microskills. *Administration and Policy in Mental Health*, *45*(5), 800–812. https://doi.org/10.1007/s10488-018-0865-y | No supervisee or patient related outcomes |
| Bearman, S. K., Weisz, J. R., Chorpita, B. F., Hoagwood, K., Ward, A., Ugueto, A. M., & Bernstein, A. (2013). More practice, less preach? The role of supervision processes and therapist characteristics in EBP implementation. *Administration and Policy in Mental Health*, *40*(6), 518–529. https://doi.org/10.1007/s10488-013-0485-5 | No supervisee or patient related outcomes |
| Berg, A., & Hallberg, I. R. (1999). Effects of systematic clinical supervision on psychiatric nurses' sense of coherence, creativity, work-related strain, job satisfaction and view of the effects from clinical supervision: A pre-post test design. *Journal of Psychiatric and Mental Health Nursing*, *6*(5), 371–381. https://doi.org/10.1046/j.1365-2850.1999.00235.x | Wrong participant characteristics |
| Berg, K. S., & Stone, G. L. (1980). Effects of conceptual level and supervision structure on counselor skill development. *Journal of Counseling Psychology*, 27(5), 500–509. https://doi.org/10.1037/0022-0167.27.5.500 | Wrong intervention |
| Bernstein, B. L., & Lecomte, C. (1979). Supervisory-type feedback effects: Feedback discrepancy level, trainee psychological differentiation, and immediate responses. *Journal of Counseling Psychology, 26*(4), 295–303. https://doi.org/10.1037/0022-0167.26.4.295 | Wrong intervention |
| Blackman, J. S. (2025). Supervision of psychoanalytic therapies based on the professional development of the supervisee. *American Journal of Psychoanalysis*, *85*(1), 108–126. https://doi.org/10.1057/s11231-025-09491-z | Wrong study design |
| Boëthius, S. B., & Ögren, M.‑L. (2001). Role Patterns in Group Supervision. *The Clinical Supervisor*, *19*(2), 45–69. https://doi.org/10.1300/J001v19n02_03 | No supervisee or patient related outcomes |
| Boëthius, S. B., Ögren, M.‑L., Sjøvold, E., & Sundin, E. C. (2004). Experiences of group culture and patterns of interaction in psychotherapy supervision groups. *The Clinical Supervisor*, *23*(1), 101–120. https://doi.org/10.1300/J001v23n01_07 | No supervisee or patient related outcomes |
| Boëthius, S. B., Sudin, E. V., & Ögren, M.‑L. (2006). Group supervision from a small group perspective. *Nordic Psychology*, *58*(1), 22–42. https://doi.org/10.1027/1901-2276.58.1.22 | Wrong intervention |
| Bowles, N., & Young, C. (1999). An evaluative study of clinical supervision based on Proctor's three function interactive model. *Journal of advanced nursing*, 30(4), 958–964. https://doi.org/10.1046/j.1365-2648.1999.01179.x | Wrong study design |
| Caballero-Suárez, N. P., Gálvez-Hernández, C. L., Huerta-Barrientos, C., Monroy-Ortiz, I. C., Sánchez-Ramos, S. M., & Sánchez-Sosa, J. J. (2023). Programa de supervisión clínica en línea para terapeutas cognitivo-conductuales en contextos hospitalarios: un estudio piloto. *Psicología Y Salud*, *33*(2), 427–439. https://doi.org/10.25009/pys.v33i2.2825 | Language not english or german |
| Callahan, J. L., Almstrom, C. M., Swift, J. K., Borja, S. E., & Heath, C. J. (2009). Exploring the contribution of supervisors to intervention outcomes. *Training and Education in Professional Psychology*, *3*(2), 72–77. https://doi.org/10.1037/a0014294 | Wrong study design |
| Calvert, F. L., Deane, F. P., & Barrett, J. (2020). Improvements in psychologists' metacommunication self-efficacy, willingness, and skill following online training and a supervision exercise. *Journal of Clinical Psychology*, *76*(6), 1083–1100. https://doi.org/10.1002/jclp.22889 | Wrong intervention |
| Calvert, F. L., Deane, F. P., & Barrett, J. (2020). Supervisees' experiences of a metacommunication intervention in clinical supervision. *Clinical Psychologist*, *24*(1), 30–40. https://doi.org/10.1111/cp.12199 | Wrong study design |
| Cox, D. L., & Araoz, G. (2009). The experience of therapy supervision within a UK multi‐centre randomized controlled trial. *Learning in Health and Social Care*, *8*(4), 301–314. https://doi.org/10.1111/j.1473-6861.2009.00218.x | Wrong study design |
| Cox, D., Burgess, M., Chalder, T., Sharpe, M., White, P., & Clark, L. (2013). Training, supervision and therapists’ adherence to manual-based therapy. *International Journal of Therapy and Rehabilitation*, *20*(4), 180–186. https://doi.org/10.12968/ijtr.2013.20.4.180 | No supervisee or patient related outcomes |
| Davis-Wright, J. O., McMahon, S. D., Miller, S. A., & Vas, S. N. (2025). Exploring the network structure of a measure of supervision competence and its prediction of trainee development. *Training and Education in Professional Psychology*, *19*(2), 106–115. https://doi.org/10.1037/tep0000506 | Wrong study design |
| DelTosta, J. E., Ellis, M. V., & McNamara, M. L. (2019). Trainee vicarious traumatization: Examining supervisory working alliance and trainee empathy. *Training and Education in Professional Psychology*, *13*(4), 300–306. https://doi.org/10.1037/tep0000232 | Wrong study design |
| DePue, M. K., Liu, R., Lambie, G. W., & Gonzalez, J. (2022). Examining the effects of the supervisory relationship and therapeutic alliance on client outcomes in novice therapists. *Training and Education in Professional Psychology*, *16*(3), 253–262. https://doi.org/10.1037/tep0000320 | Wrong study design |
| Drinane, J. M., Wilcox, M. M., Cabrera, L., & Black, S. W. (2021). To conceal or not to conceal: Supervisee and client identity processes in clinical supervision. *Psychotherapy (Chicago, Ill.)*, *58*(4), 429–436. https://doi.org/10.1037/pst0000387 | Wrong study design |
| Ellis, M. V. (2006). Critical incidents in clinical supervision and in supervisor supervision: Assessing supervisory issues. *Training and Education in Professional Psychology*, *S*(2), 122–132. https://doi.org/10.1037/1931-3918.S.2.122 | No supervisee or patient related outcomes |
| Ellis, M. V., Hutman, H., & Chapin, J. (2015). Reducing supervisee anxiety: Effects of a role induction intervention for clinical supervision. *Journal of Counseling Psychology*, *62*(4), 608–620. https://doi.org/10.1037/cou0000099 | Wrong intervention |
| Evers, O., Schröder-Pfeifer, P., Möller, H., & Taubner, S. (2019). How do personal and professional characteristics influence the development of psychotherapists in training: Results from a longitudinal study. *Research in Psychotherapy (Milano)*, *22*(3), 424. https://doi.org/10.4081/ripppo.2019.424 | Wrong intervention |
| Fleming, L. M., Glass, J. A., Fujisaki, S., & Toner, S. L. (2010). Group process and learning: A grounded theory model of group supervision. *Training and Education in Professional Psychology*, *4*(3), 194–203. https://doi.org/10.1037/a0018970 | Wrong study design |
| Geisler Forshammar, C., Geisler, M., & Buratti, S. (2025). The moderating role of clinical supervision on the relationship between emotional demands and exhaustion among clinical psychologists in Sweden. *The Clinical Supervisor*, *44*(1), 77–98. https://doi.org/10.1080/07325223.2024.2442986 | Wrong study design |
| Gillam, R. B., Roussos, C. S., & Anderson, J. L. (1990). Facilitating changes in supervisees' clinical behaviors: An experimental investigation of supervisory effectiveness. *The Journal of Speech and Hearing Disorders*, *55*(4), 729–739. https://doi.org/10.1044/jshd.5504.729 | Wrong participant characteristics |
| Gonge, H., & Buus, N. (2011). Model for investigating the benefits of clinical supervision in psychiatric nursing: A survey study. *International Journal of Mental Health Nursing*, *20*(2), 102–111. https://doi.org/10.1111/j.1447-0349.2010.00717.x | Wrong participant characteristics |
| Gonge, H., & Buus, N. (2015). Is it possible to strengthen psychiatric nursing staff's clinical supervision? RCT of a meta-supervision intervention. *Journal of Advanced Nursing*, *71*(4), 909–921. https://doi.org/10.1111/jan.12569 | Wrong intervention |
| Goodyear, R. K., & Sera, H. (2024). Supervisees’ perspectives on countertransference-focused practices and processes in clinical supervision: an exploratory study. *The Clinical Supervisor*, *43*(2), 269–290. https://doi.org/10.1080/07325223.2024.2362127 | Wrong study design |
| Heatley, C., Ricketts, T., & Forrest, J. (2005). Training general practitioners in cognitive behavioural therapy for panic disorder: Randomized-controlled trial. *Journal of Mental Health*, *14*(1), 73–82. https://doi.org/10.1080/09638230500047877 | Wrong intervention |
| Heaven, C., Clegg, J., & Maguire, P. (2006). Transfer of communication skills training from workshop to workplace: The impact of clinical supervision. *Patient Education and Counseling*, *60*(3), 313–325. https://doi.org/10.1016/j.pec.2005.08.008 | Wrong participant characteristics |
| Hedegaard, A. E. (2020). The supervisory alliance in group supervision. *British Journal of Psychotherapy*, *36*(1), 45–60. https://doi.org/10.1111/bjp.12495 | No supervisee or patient related outcomes |
| Hendricks, S., Cartwright, D. J., & Cowden, R. G. (2021). Clinical supervision in South Africa: Perceptions of supervision training, practices, and professional competencies. *South African Journal of Science*, *117*(3/4). https://doi.org/10.17159/sajs.2021/7428 | Wrong intervention |
| Henrich, D., Glombiewski, J. A., & Scholten, S. (2025). Supervisees’ evaluations of a structured video-supervision technique. *Cognitive Behaviour Therapist*, *18*(e25). https://doi.org/10.1017/s1754470x25000145 | No supervisee or patient related outcomes |
| Hiebler-Ragger, M., Nausner, L., Blaha, A., Grimmer, K., Korlath, S., Mernyi, M., & Unterrainer, H. F. (2021). The supervisory relationship from an attachment perspective: Connections to burnout and sense of coherence in health professionals. *Clinical Psychology & Psychotherapy*, *28*(1), 124–136. https://doi.org/10.1002/cpp.2494 | Wrong study design |
| Hill, H. R. M., Crowe, T. P., & Gonsalvez, C. J. (2016). Reflective dialogue in clinical supervision: A pilot study involving collaborative review of supervision videos. *Psychotherapy Research: Journal of the Society for Psychotherapy Research*, *26*(3), 263–278. https://doi.org/10.1080/10503307.2014.996795 | Wrong study design |
| Hilsenroth, M. J., Kivlighan, D. M., Jr, & Slavin-Mulford, J. (2015). Structured supervision of graduate clinicians in psychodynamic psychotherapy: alliance and technique. *Journal of counseling psychology*, *62*(2), 173–183. https://doi.org/10.1037/cou0000058 | Wrong study design |
| Hogue, A., MacLean, A., Bobek, M., Dunnsue, S., Porter, N., Jensen-Doss, A., & Henderson, C. E. (2025). Training community therapists in core elements of CBT and family therapy for adolescent externalizing problems. *Journal of Clinical Child and Adolescent Psychology, Division 53*, *54*(2), 255–271. https://doi.org/10.1080/15374416.2023.2222405 | Wrong intervention |
| Hunt, C., & Sharpe, L. (2008). Within-session supervision communication in the training of clinical psychologists. *Australian Psychologist*, *43*(2), 121–126. https://doi.org/10.1080/00050060801933402 | Wrong study design |
| Jakob, M., Weck, F., & Bohus, M. (2013). Live-Supervision: Vom Einwegspiegel zur videobasierten Online-Supervision. *Verhaltenstherapie*, *23*(3), 170–180. https://doi.org/10.1159/000354234 | No supervisee or patient related outcomes |
| Johnson, J., Corker, C., & O'connor, D. B. (2020). Burnout in psychological therapists: A cross‐sectional study investigating the role of supervisory relationship quality. *Clinical Psychologist*, *24*(3), 223–235. https://doi.org/10.1111/cp.12206 | Wrong study design |
| Johnson, J. E., Hailemariam, M., Zlotnick, C., Richie, F., & Wiltsey-Stirman, S. (2024). Analysis of implementation processes in a hybrid effectiveness-implementation trial of interpersonal psychotherapy (IPT) for major depressive disorder in prisons: Training, supervision, and recommendations. *PloS One*, *19*(5), e0288182. https://doi.org/10.1371/journal.pone.0288182 | Wrong study design |
| Kang, S., & Yu, K. (2025). An experimental study of multicultural orientation in clinical supervision in South Korea: Supervisees’ perceptions of supervision processes and outcomes. *Professional Psychology: Research and Practice*, *56*(2), 172–180. https://doi.org/10.1037/pro0000601 | Wrong study design |
| Kallander, K., Tibenderana, J., Kirkwood, B., Hill, Z., Strachan, D., Soremekun, S., Lingam, R., Vassal, A., Kasteng, F., & Meek, S. (2012). Inscale cluster randomized trial evaluating the effect of innovative motivation and supervision approaches on community health worker performance and retention in Uganda and Mozambique: intervention design, *87*(5), 243. https://www.cochranelibrary.com/central/doi/10.1002/central/CN-01027615/full | Wrong study design |
| Keenan-Miller, D., & Corbett, H. I. (2015). Metasupervision: Can students be safe and effective supervisors? *Training and Education in Professional Psychology*, *9*(4), 315–321. https://doi.org/10.1037/tep0000090 | Wrong intervention |
| Kennard, B. D., Stewart, S. M., & Gluck, M. R. (1987). The supervision relationship: Variables contributing to positive versus negative experiences. *Professional Psychology: Research and Practice*, *18*(2), 172–175. https://doi.org/10.1037/0735-7028.18.2.172 | No supervisee or patient related outcomes |
| Khani A., Jaafarpour M., & Jamshidbeigi Y. (2008). The relationship between clinical supervision and burnout in the nurse's job - An Iranian study, *2*(4), 913–918. http://www.jcdr.net/articles/PDF/304/280_E(c)_F(P)_R(p)_Pf_p.pdf | Wrong study design |
| Kivimäki, M. (1996). Confidential conversations between supervisor and employee as a means for improving leadership: a quasi-experimental study in hospital wards. *Journal of Nursing Management*, *4*(6), 325–335. https://doi.org/10.1046/j.0966-0429.2001.00294.x-i1 | Wrong participant characteristics |
| Knox, S., Burkard, A. W., Edwards, L. M., Smith, J. J., & Schlosser, L. Z. (2008). Supervisors' reports of the effects of supervisor self-disclosure on supervisees. *Psychotherapy Research: Journal of the Society for Psychotherapy Research*, *18*(5), 543–559. https://doi.org/10.1080/10503300801982781 | Wrong study design |
| Kovač, J., Krečič, M. J., Čagran, B., & Mulej, M. (2017). Effect of Supervision on Stress and Burnout in School Counsellors: A Case of Action Research. *Systemic Practice and Action Research*, *30*(4), 395–406. https://doi.org/10.1007/s11213-016-9400-9 | Wrong participant characteristics |
| Ladany, N., Mori, Y., & Mehr, K. E. (2013). Effective and Ineffective Supervision. *The Counseling Psychologist*, *41*(1), 28–47. https://doi.org/10.1177/0011000012442648 | Wrong study design |
| Liddell, A. E., Allan, S., & Goss, K. (2017). Therapist competencies necessary for the delivery of compassion-focused therapy: A Delphi study. *Psychology and Psychotherapy*, *90*(2), 156–176. https://doi.org/10.1111/papt.12105 | Wrong study design |
| Livni, D., Crowe, T. P., & Gonsalvez, C. J. (2012). Effects of supervision modality and intensity on alliance and outcomes for the supervisee. *Rehabilitation Psychology*, *57*(2), 178–186. https://doi.org/10.1037/a0027452 | Wrong participant characteristics |
| Lizzio, A., Wilson, K., & Que, J. (2009). Relationship dimensions in the professional supervision of psychology graduates: supervisee perceptions of processes and outcome. *Studies in Continuing Education*, *31*(2), 127–140. https://doi.org/10.1080/01580370902927451 | Wrong study design |
| Locke, J., Violante, S., Pullmann, M. D., Kerns, S. E. U., Jungbluth, N., & Dorsey, S. (2018). Agreement and discrepancy between supervisor and clinician alliance: Associations with clinicians' perceptions of psychological climate and emotional exhaustion. *Administration and Policy in Mental Health*, *45*(3), 505–517. https://doi.org/10.1007/s10488-017-0841-y | Wrong study design |
| Lyon, A. R., Dorsey, S., Pullmann, M., Silbaugh-Cowdin, J., & Berliner, L. (2015). Clinician use of standardized assessments following a common elements psychotherapy training and consultation program. *Administration and Policy in Mental Health*, *42*(1), 47–60. https://doi.org/10.1007/s10488-014-0543-7 | Wrong intervention |
| MacCulloch, T., & Shattell, M. (2009). Clinical supervision and the well-being of the psychiatric nurse. *Issues in Mental Health Nursing*, *30*(9), 589–590. https://doi.org/10.1080/01612840902954541 | Wrong study design |
| Malikiosi-Loizos, M., Mehnert, W. O., Work, G. G., & Gold, J. (1981). Differential supervision and cognitive structure effects on empathy and counseling effectiveness. *International Journal for the Advancement of Counselling*, *4*(2), 119–129. https://doi.org/10.1007/BF00139735 | Wrong participant characteristics |
| Marrow, C. E., Hollyoake, K., Hamer, D., & Kenrick, C. (2002). Clinical supervision using video-conferencing technology: a reflective account. *Journal of nursing management*, *10*(5), 275–282. https://doi.org/10.1046/j.1365-2834.2002.00313.x | Wrong study design |
| Martino, S., Zimbrean, P., Forray, A., Kaufman, J. S., Desan, P. H., Olmstead, T. A., Gilstad-Hayden, K., Gueorguieva, R., & Yonkers, K. A. (2019). Implementing motivational interviewing for substance misuse on medical inpatient units: A randomized controlled trial. *Journal of General Internal Medicine*, *34*(11), 2520–2529. https://doi.org/10.1007/s11606-019-05257-3 | Wrong intervention |
| McCutcheon, K., O'Halloran, P., & Lohan, M. (2018). Online learning versus blended learning of clinical supervisee skills with pre-registration nursing students: A randomised controlled trial. *International Journal of Nursing Studies*, *82*, 30–39. https://doi.org/10.1016/j.ijnurstu.2018.02.005 | Wrong participant characteristics |
| Meier, A., McGovern, M. P., Lambert-Harris, C., McLeman, B., Franklin, A., Saunders, E. C., & Xie, H. (2015). Adherence and competence in two manual-guided therapies for co-occurring substance use and posttraumatic stress disorders: Clinician factors and patient outcomes. *The American Journal of Drug and Alcohol Abuse*, *41*(6), 527–534. https://doi.org/10.3109/00952990.2015.1062894 | Wrong intervention |
| Meier, A., McGovern, M. P., Lambert-Harris, C., McLeman, B., & Saunders, E. (2017). A pilot trial of two models of clinical supervision of integrated cognitive behavioral therapy for PTSD and substance use disorders. *Drug and Alcohol Dependence*, *171*, e141. https://doi.org/10.1016/j.drugalcdep.2016.08.391 | Not peer-reviewed |
| Miljkovic, E. (2023). Comparing presence and absence of initial in-person contact and written feedback in RE&CBT e-supervision. *Journal of Rational-Emotive and Cognitive-Behavior Therapy*, 1–33. https://doi.org/10.1007/s10942-023-00505-2 | No supervisee or patient related outcomes |
| Milne, D. L., & James, I. A. (2002). The observed impact of training on competence in clinical supervision. *British Journal of Clinical Psychology*, *41*(Pt 1), 55–72. https://doi.org/10.1348/014466502163796 | Wrong intervention |
| Moran, A., Shanahan, A., Tomlin, A., Ivers, R., & Thomas, S. J. (2023). Pilot study of a group clinical supervision model for medical students. *Australasian Psychiatry: Bulletin of Royal Australian and New Zealand College of Psychiatrists*, *31*(5), 694–699. https://doi.org/10.1177/10398562231186238 | Wrong participant characteristics |
| Newman, D. S., Villarreal, J. N., Gerrard, M. K., McIntire, H., Barrett, C. A., & Kaiser, L. T. (2022). Deliberate practice of consultation communication skills: A randomized controlled trial. *School Psychology (Washington, D.C.)*, *37*(3), 225–235. https://doi.org/10.1037/spq0000494 | Wrong intervention |
| North, G. J. (2013). Recording supervision: Educational, therapeutic, and enhances the supervisory working alliance? *Counselling and Psychotherapy Research*, *13*(1), 61–70. https://doi.org/10.1080/14733145.2012.687386 | Wrong study design |
| Nyman, S. J., Nafziger, M. A., & Smith, T. B. (2010). Client outcomes across counselor training level within a multitiered supervision model. *Journal of Counseling & Development*, *88*(2), 204–209. https://doi.org/10.1002/j.1556-6678.2010.tb00010.x | No supervisee or patient related outcomes |
| Ochs, P. C. (2021). Using action methods in clinical supervision: a journey from talk to action. *Social Work with Groups*, *44*(3), 258–272. https://doi.org/10.1080/01609513.2020.1793057 | Wrong study design |
| O'Connor, D. W., & Spratt, C. (2010). Expanded specialist training: Psychiatry supervisors' feedback. *Australasian Psychiatry: Bulletin of Royal Australian and New Zealand College of Psychiatrists*, *18*(3), 268–269. https://doi.org/10.3109/10398561003731205 | Wrong study design |
| Ögren, M.‑L., Jonsson, C.‑O., & Sundin, E. C. (2005). Group supervision in psychotherapy: The relationship between focus, group climate, and perceived attained skill. *Journal of Clinical Psychology*, *61*(4), 373–388. https://doi.org/10.1002/jclp.20056 | Wrong study design |
| O'Keeffe, F., Watson, S., & Linke, S. (2016). Training novice clinical psychologist trainees to implement effective CBT for anxiety disorders: training model and clinic outcomes. *The Cognitive Behaviour Therapist*, *9.* https://doi.org/10.1017/S1754470X16000246 | Wrong intervention |
| O'Mahony, E., & Corvin, A. (2001). The attitudes of Irish trainees to their training and its supervision: A five-year follow up study. *Irish Journal of Psychological Medicine*, *18*(4), 120–125. https://doi.org/10.1017/S0790966700006595 | Wrong study design |
| Ooijen van, E., & Spencer, L. (2017). Practitioners’ perspectives on how supervision training has impacted their practice. *Counselling and Psychotherapy Research*, *17*(4), 283–290. https://doi.org/10.1002/capr.12128 | Wrong study design |
| Öztürk, F., & Duran, N. O. (2024). The mediating role of the evaluation process within supervision on the relationship between counseling self-efficacy and working alliance. *International Journal for the Advancement of Counselling*, *46*(1), 20–39. https://doi.org/10.1007/s10447-023-09526-z | Wrong study design |
| Probst, T., Jakob, M., Kaufmann, Y. M., Müller-Neng, J. M. B., Bohus, M., & Weck, F. (2018). Patients' and therapists' experiences of general change mechanisms during bug-in-the-eye and delayed video-based supervised cognitive-behavioral therapy. A randomized controlled trial. *Journal of Clinical Psychology*, *74*(4), 509–522. https://doi.org/10.1002/jclp.22519 | Wrong study design |
| Rahman, A., Akhtar, P., Hamdani, S. U., Atif, N., Nazir, H., Uddin, I., Nisar, A., Huma, Z., Maselko, J., Sikander, S., & Zafar, S. (2019). Using technology to scale-up training and supervision of community health workers in the psychosocial management of perinatal depression: A non-inferiority, randomized controlled trial. *Global Mental Health*, *6*, e8. https://doi.org/10.1017/gmh.2019.7 | Wrong intervention |
| Rocha, T. I. U., Aschar, S. C. d. A. L., Hidalgo-Padilla, L., Daley, K., Claro, H. G., Martins Castro, H. C., Dos Santos, D. V. C., Miranda, J. J., Araya, R., & Menezes, P. R. (2021). Recruitment, training and supervision of nurses and nurse assistants for a task-shifting depression intervention in two RCTs in Brazil and Peru. *Human Resources for Health*, *19*(1), 16. https://doi.org/10.1186/s12960-021-00556-5 | Wrong participant characteristics |
| Rønnestad, M. H., Orlinsky, D. E., & Willutzki, U. (2025). Exploring influences of supervision on psychotherapists’ professional development: correlates across career-level cohorts. *Counselling Psychology Quarterly*, *38*(2), 297–319. https://doi.org/10.1080/09515070.2024.2378879 | Wrong study design |
| Rousmaniere, T. G., Swift, J. K., Babins-Wagner, R., Whipple, J. L., & Berzins, S. (2016). Supervisor variance in psychotherapy outcome in routine practice. *Psychotherapy Research: Journal of the Society for Psychotherapy Research*, *26*(2), 196–205. https://doi.org/10.1080/10503307.2014.963730 | Wrong study design |
| Rowen, J., Giedgowd, G., & Baran, D. (2022). Effective and accessible telephone-based psychotherapy and supervision. *Journal of Psychotherapy Integration*, *32*(1), 3–18. https://doi.org/10.1037/int0000257 | No supervisee or patient related outcomes |
| Ryu, H., & Hamilton, B. (2023). Evaluation of clinical supervision implementation for mental health nurses in Victoria, Australia. *International Journal of Mental Health Nursing*, *32*, 60–61. | Not peer-reviewed |
| Schmidt, L. M., & Foli-Andersen, N. J. (2017). Psychotherapy and cognitive behavioral therapy supervision in Danish psychiatry: Training the Next Generation of Psychiatrists. *Academic Psychiatry: The Journal of the American Association of Directors of Psychiatric Residency Training and the Association for Academic Psychiatry*, *41*(1), 4–9. https://doi.org/10.1007/s40596-015-0442-6 | Wrong study design |
| Šefarová, I., & Šlepecký, M. (2017). Self-efficacy of the psychotherapist in the context of supervision. *European Psychiatry*, *41*(S1), s506-s506. https://doi.org/10.1016/j.eurpsy.2017.01.645 | Not peer-reviewed |
| Sobell, L. C., Manor, H. L., Sobell, M. B., & Dum, M. (2008). Self-critiques of audiotaped therapy sessions: A motivational procedure for facilitating feedback during supervision. *Training and Education in Professional Psychology*, *2*(3), 151–155. https://doi.org/10.1037/1931-3918.2.3.151 | No supervisee or patient related outcomes |
| Steel, C., Tarrier, N., Stahl, D., & Wykes, T. (2012). Cognitive behaviour therapy for psychosis: The impact of therapist training and supervision. *Psychotherapy and Psychosomatics*, *81*(3), 194–195. https://doi.org/10.1159/000334250 | Wrong study design |
| Szeftel, R., Hakak, R., Meyer, S., Naqvi, S., Sulman-Smith, H., Delrahim, K., & Rapaport, M. (2008). Training psychiatric residents and fellows in a telepsychiatry clinic: A supervision model. *Academic Psychiatry*, *32*(5), 393–399. https://doi.org/10.1176/appi.ap.32.5.393 | Wrong participant characteristics |
| Tracey, T. J. G., Bludworth, J., & Glidden-Tracey, C. E. (2012). Are there parallel processes in psychotherapy supervision? An empirical examination. *Psychotherapy (Chicago, Ill.)*, *49*(3), 330–343. https://doi.org/10.1037/a0026246 | No supervisee or patient related outcomes |
| Tsegos, I. K., Karayanni, V., Karapostoli, N., & Morarou, E. (2004). An extensive outcome of a new approach to supervision: a research study derived from supervision protocols of students' psychotherapeutic and psychometric activities. *European Journal of Psychiatry*, *18*, 61–70. | No supervisee or patient related outcomes |
| Vannucci, M. J., Whiteside, D. M., Saigal, S., Nichols, L., & Hileman, S. (2017). Predicting Supervision Outcomes: What is Different about Psychological Assessment Supervision? *Australian Psychologist*, *52*(2), 114–120. https://doi.org/10.1111/ap.12258 | Wrong participant characteristics |
| Wells, R., Acartuk, C., Almeamari, F., Alokoud, M., Beetar, A., Eldardery, H., Elshazly, M., Faruk, O., Ginem, M. R., Hadzi-Pavlovic, D., Ilkkurşun, Z., Jahan, S., Joshi, R., Klein, L., Kurdi, L., Kurt, G., Mastrogiovanni, C., Mozumder, M., Lekkeh, S., . . . Rosenbaum, S. (2022). Caring for carers: A virtual psychosocial supervision intervention to improve the quality and sustainability of mental health and psychosocial support in humanitarian contexts. *European Psychiatry*, | Wrong study design |
| Whipple, J., Hoyt, T., Rousmaniere, T., Swift, J., Pedersen, T., & Worthen, V. (2020). Supervisor variance in psychotherapy outcome in routine practice: A replication. *SAGE Open*, *10*(1), 215824401989904. https://doi.org/10.1177/2158244019899047 | Wrong study design |
| White, E. (2010). FC03-05 - quality of care and patient outcomes: A randomised trial of clinical supervision in mental health settings in Queensland, Australia. *European Psychiatry*, *25*(S1), 1–1. https://doi.org/10.1016/s0924-9338(10)70191-7 | Wrong study design |
| White, E., & Winstanley, J. (2009). Clinical supervision for nurses working in mental health settings in Queensland, Australia: a randomised controlled trial in progress and emergent challenges. *Journal of Research in Nursing*, *14*(3), 263–276. https://doi.org/10.1177/1744987108101612 | Wrong study design |
| White, Edward, & Winstanley, J. (2010). A randomised controlled trial of clinical supervision: selected findings from a novel Australian attempt to establish the evidence base for causal relationships with quality of care and patient outcomes, as an informed contribution to mental health nursing practice development. *Journal of Research in Nursing*, *15*(2), 151–167. https://doi.org/10.1177/1744987109357816 | Wrong participant characteristics |
| White, E., & Winstanley, J. (2010). Does clinical supervision lead to better patient outcomes in mental health nursing? *Nursing times*, *106*(16), 16–18. | Wrong participant characteristics |

# Appendix D Overview of included studies

| **Study (Design)** | **Country** | **Supervisors‘ characteristics** | **Supervisees‘ characteristics** | **Study-reported supervision type and characteristics** | **Therapy and patients’ characteristics** |
| --- | --- | --- | --- | --- | --- |
| Alfonsson et al., 2020  (single-case experimental multiple baseline design) | Sweden | 1 supervisor (advanced training in CBT), several years of experience with clinical supervision | 6 therapists (4 psychologists, 2 counselors)  66.7% female  Age (m) = 33.1  Clinical exp. (m) = 2.8 y | 5 to 8 sessions of 50-60 minutes CBT supervision  Competence-focused supervision instead of case-focused supervision, i.e. feedback was provided regarding the chosen focus items, and not the respective case | 15 patients included |
| Anderson et al., 2012  (uncontrolled) | USA | Authors of time-limited dynamic psychotherapy (TLDP) | 16 therapists (8 psychologists, 8 psychiatrists); psychodynamic psychotherapy approach  37.5% female  Clinical exp. (m) = 4.3 y | Group supervision (length (m) = 53.5 min) | 48 patients included (72.9% female, age (m) = 40.7)  time-limited dynamic psychotherapy (TLDP); 22 sessions in 25 weeks |
| Andersson et al., 2023  (controlled, factorial design 2x2x2x3) | Sweden | Psychologist, expert on ICBT | 6 therapists (M. Sc. Psychology Students) | One factor: Supervision available vs. supervision not available  INT: 8 supervision sessions over 8 weeks  CG: No supervision | 197 patients with depression included (INT: 73.7% female, age (m) = 34,41; CG: 80.6%, age (m) = 34,83),  ICBT; 10 weekly sessions recommended |
| Bambling et al., 2006 (RCT) | USA | 40 supervisors, 77.5% female, Supervision experience (m) = 11.2 y | 103 therapists (6 doctorate degree, 32 registration-level qualification in psychology; 27 master’s degree in psychology, 27 master’s degree in psychotherapy, 9 master’s degree in social work, and 6 social work degree; 20 graduates in other mental health disciplines)  INT: N = 34, 82.4% female  Age (m) = 41.7  Clinical exp. (m) = 8.6 y  AC: N = 33, 66.7% female  Age (m) = 44.7  Clinical exp. (m) = 9 y  CG: N = 60, 61.7% female  Age (m) = 45.2  Clinical exp. (m) = 8.8 y | 8 weeks of supervision  INT: Process-focus supervision  AC: Skill-focus supervision  CG: No supervision | 103 patients included with depression  Problem-solving therapy (PST, form of CBT); 8 weekly sessions |
| Bartle-Haring et al., 2009  (uncontrolled) | USA | No information provided | 10 therapists, 70% female  clinical exp. (m) = 1.4 years | 1-3 sessions of live supervision, supervision in variable forms (video-based, live, case-report) and variable frequencies  Total: 0-6 sessions of supervision | 394 cases (52.4% female, age (m) = 31.302)  6 sessions of marriage and family therapy |
| Bearman et al., 2017  (RCT) | USA | 28 supervisors | 40 therapists (18 doctoral programs in clinical psychology, 10 school-clinical child psychology, 4 master’s in social work, 8 masters in mental health counselling)  INT: N = 21, 90.5% female  Age (m) = 25.05  Clinical exp. (m) = 1.23 y  CG: N = 19, 89.5% female  Age (m) = 25,42  Clinical exp. (m) = 1.58 y  Theoretical orientation: 7 psychodynamic, 20 behavioral/CBT, 11 other/integrated | 3 weekly 60-minutes group supervision sessions  INT: Supervision + (scaffolding and experiential learning)  CG: SAU | 4 standardized vignettes of 12-year-old girls played by 4 young female research assistants. |
| Beidas et al., 2012  (uncontrolled for supervision condition) | USA | No information provided | 115 therapists (community therapists with various academic degrees), 90.4% female  Age (m) = 35.93  Clinical exp. (m) = 5.46 y  Theoretical orientation: CBT | 12 weeks computer- or telephone-based group supervision (length (m) = 52.6 min, participants (m) = 7.8) | Therapy for anxiety disorder in children |
| Bradshaw et al., 2007  (controlled) | UK | Mental health nurses with a 2-day course in clinical supervision | 23 therapists (mental health nurses in PSI-programme)  INT: N = 11, 45.5% female  Age (m) = 36.6  Clinical exp. (m) = 9.8 y  CG: N = 12, 75.0% female  Age (m) = 31.8  Clinical exp. (m) = 5.8 y | Biweekly group supervision sessions (2 supervisees, 1 supervisor; length between 60 and 90 min)  INT: Workplace supervision  CG: No workplace supervision | 93 patients with schizophrenia (INT: N = 38, CG: N = 55) |
| Carmel et al., 2016  (RCT) | USA | 5 supervisors  (1 BITE Supervisor highly trained in DBT, less than 10 years clinical experience; 4 SAU supervisors, >10 years of experience in DBT) | 8 psychiatrists in training  (37.5% cognitive behavioral,  12.5% psychoanalytic,  25% other psychodynamic,  12.5% Gestalt, 12.5% eclectic)  INT: N = 4  CG: N = 4 | 9 months of weekly supervision  INT: Live supervision (BITE; group)  CG: SAU (individual) | Dialectical behavior therapy during psychiatrists’ 11-month rotation |
| Dorsey et al., 2025  (RCT)^1^ | USA | 48 supervisors (CBT specific training) | 130 therapists | CBT supervision focused on the delivery of trauma-focused cognitive behavioral therapy (TF-CBT)  INT: Symptom and fidelity monitoring + Behavioral Rehearsal  CG: Symptom and fidelity monitoring | 206 patients; trauma-exposed youth  TF-CBT |
| Ellis et al., 2002  (“study 2”, RCT) | USA | 1 supervisor (4 years of supervision experience), male | 81 counsellors, 70.4% female, age (m) = 27.72 y  (graduate students in counseling psychology, clinical psychology, social work;  48.1% CBT, 23.5% eclectic, 17.3% psychodynamic, 11.1% other)  INT: N = 27  AC: N = 27  CG: N = 27 | INT: Public self-awareness, counselling session was videotaped and reviewed with a supervisor for 20 minutes  AC: Private self-awareness, counselling sessions was audio taped and reviewed by themselves for 20 minutes  CG: Subjective awareness: no aversive effects of audio- or videotaping, counselling sessions was reviewed with a patient summary sheet for 5 minutes | 20-minute counseling session with a standardized angry patient |
| Johnson et al., 2023  (uncontrolled) | Canada | 15 supervisors (12 social workers, 3 doctoral level psychologists) | 35 social workers (CBT) | 10 sessions of 60-min supervision  (2- day CBT workshop before supervision) | 45 children and adolescents |
| Karlsruher, 1976  (RCT) | Canada | 2 supervisors (graduate students with a master's in clinical psychology), at least 1 year of experience doing supervised psychotherapy | 20 undergraduate students  INT: N = 10  CG: N = 10 | 10 sessions of 60-min weekly group supervision (group of 5 supervisees)  INT: Supervision available  CG: No supervision available | 5^th^ and 6^th^ grade boys  Weekly 45-min patient centered therapy sessions for 10 weeks |
| Kivlighan et al., 1991  (controlled) | USA | 9 supervisiors (1 doctoral-level counselling psychologist, 8 advanced doctoral-level counselling psychology students) | 48 counsellors in training  INT: N = 23, 60.9% female  Age (m) = 26.0  CG: N = 25, 60% female  Age (m) = 25.8 | 4 sessions group supervision  INT: Live supervision for 80 minutes  CG: Video-taped supervision for 60 minutes | 48 patients (INT: N = 23, 82% female, age (m) = 21.3; CG: N = 25, 78% female, age (m) = 21.5); with a personal concern (interpersonal conflicts, depressed moods, loneliness, difficulties with assertiveness, low self-esteem)  Interpersonal Psychotherapy (IPT);  4 sessions for 50 Minutes |
| Krall & Fürst, 2011 (uncontrolled) | Austria | 1 supervisor (clinical psychologist, experienced supervisor), female | data of 13 psychotherapists in training, 76.9% female | 6 months of group supervision (group of 3-5 supervisees) every 2-4 weeks for 3-4.5 hours  Psychodrama supervision (scenic and sociometric methods) | No information provided |
| Krall, 2022  (controlled) | Austria | 5 supervisors, 60% female | 19 psychotherapists in training, 100% female | 4-6 hours of group supervision  INT: Psychodramatic and sociometric supervision  CG: Verbal supervision (SAU) | 207 patients |
| Maaß et al., 2024  (RCT) | Germany | 1 supervisor (licensed CBT-psychotherapist), 9 years clinical experience, no previous experience as supervisor | 69 bachelor’s and master’s students  INT: N = 37, 88.2% female  Age (m) = 24.94  CG: N = 36, 77.1% female  Age (m) = 24.91 | One session of 20-minutes supervision  INT: Live supervision (BITE)  CG: No supervision | standardized patient (played by 9 different students)  20 minutes CBT-therapy session |
| Maaß et al., 2025 (RCT) | Germany | 1 supervisor (licensed CBT-psychotherapist), female, 11 years clinical experience, 12 years experience as supervisor | 80 bachelor’s and master’s students  INT: N = 39, 71.8% female  Age (m) = 23.95  CG: N = 41, 73.2% female  Age (m) = 24.61 | One supervision session  INT: Live supervision (BITE)  CG: Delayed supervision | standardized patient with sleep disorder (played by 11 different students)  3 sessions of 15 minutes CBT-therapy |
| Martino et al., 2016  (RCT) | USA | 22 supervisors | 69 therapists (68% master’s, 14% bachelor’s, 8% associate’s, 9% high school, and 1% doctorate degrees)  INT: N = 35, 86% female  Age (m) = 39.2  Clinical exp. (m) = 7.5 y  CG: N = 31, 71% female  Age (m) = 43.6  Clinical exp. (m) = 10.4 y | INT: Motivational Interviewing Assessment: Supervisory Tools for Enhancing Proficiency (MIA: STEP); 6.5 (mean) sessions of 35-minutes supervision  CG: SAU; 5 (mean) sessions of 20-minutes supervision  (Both groups received 8h workshop on Motivational Interviewing before supervision) | 450 patients with substance use disorder (INT: N = 227, 33% female, age (m) = 35.5; CG: N = 223, 35% female, age (m) = 35.4)  Motivational Interviewing (CBT) |
| Meza et al., 2023  (uncontrolled) | USA | 28 supervisors (CBT specific training), 64.3% female, age (m) = 44.4 | 70 therapists (degree in 5.7% psychology, 27.1% social work, 40% counselling, 15.7% other; 51,4% licensed)  87.1% female, age (m) = 38.0  theoretical orientation: CBT, Family systems, solution-focused, humanistic, psychodynamic, play therapy, art therapy | CBT supervision focused on the delivery of TF-CBT;  post-hoc comparing supportive-directive and supportive supervision | 60 patients, 61.7% female, age (m) = 11.5;  trauma-exposed youth  TF-CBT |
| Monson et al., 2018  (RCT) | Canada | No information provided | 80 therapists (mental health therapists practicing psychotherapy)  INT: N = 30, 70% female  age (m) = 47.21  AC: N = 30, 79% female  age (m) = 48.86  CG: N = 20, 74% female  age (m) = 46.42 | 6 months weekly 60-minutes sessions of CBT group supervision per video call (group auf 4-6 supervisees)  INT: Standard + Audio Consultation (i.e. therapists presented audio recordings of their therapy session)  AC: Standard Consultation  CG: No consultation | 188 patients with PTSD  INT: N = 74, 44.8% female,  age (m) = 41.94  AC: N = 61, 48.3% female,  age (m) = 38.0  CG: N = 53, 66% female,  age (m) = 37.31  12 sessions of Cognitive Processing Therapy (CBT-framework) |
| Ng & Cheung, 2007  (uncontrolled) | China | 1 supervisor (psychiatrist), 16 years of clinical experience, 5 years of supervision experience | 10 therapists (11 social workers, 1 nurse), 66.7% female, age (m) = 38.7 | 6 months weekly group CBT supervision for Psychosis (+Training) | 10 patients with schizophrenia, age (m) = 47.6  CBT-therapy, duration (m) = 26.4 weeks, number of sessions (m) = 12.4 |
| Ögren & Jonsson, 2003  (uncontrolled) | Sweden | Psychotherapists as supervisors | 162 therapists (psychotherapists in training; psychodynamic) | 1,5 year weekly two-hour group psychodynamic supervision (group of 3 supervisees) | No information provided |
| Plantade-Gipch et al., 2021  (controlled) | France | No information provided | 30 therapists (psychotherapists; 40% integrative, 20% psychodynamic, 30% cognitive behavioral or systemics, 10% humanistic),  90% female, age (m) = 29.1, experience (m) = 1.85 y  INT: N = 15  CG: N = 15 | 5 two-hour sessions over 5 months  INT: Alliance-focused supervision  CG: No supervision | No information provided |
| Rakovshik et al., 2016  (RCT)^2^ | Russia | 1 supervisor | 61 therapists (49 psychologists, 5 psychiatrists, 7 psychiatrists–psychotherapists)  INT: N = 22  CG: N = 19 | 3 30-minutes sessions of supervision over 3 months  INT: CBT supervision via Skype  CG: Consultation Worksheet (No supervision)  Insert as a footnote: another treatment arm, without training, which is excluded in this review; both treatment arms here included a training before supervision phase. | No information about patients provided  CBT-therapy |
| Reese, Aldarondo, et al., 2009  (uncontrolled) | USA | 1 supervisor (licensed psychologist), 10 years of experience as a supervisor) | 9 therapists (counselling psychology masters and doctoral students), 88.89% female, age (m) = 29 y | 12 weeks of weekly 2.5h group supervision | No information provided |
| Reese, Usher, et al., 2009  (controlled) | USA | 9 supervisors  (degree in clinical-counseling (master's or doctorate) or marriage and family therapy) | 28 therapists (masters students), 64.3% female  INT: N = 11  CG: N = 17 | 16 weeks of weekly individual and group supervision  INT: Discussing patient feedback in supervision  (with Partners for Change Outcome Management System)  CG: No patient feedback in supervision | 110 patients, 70.9% female  with mood and anxiety disorders, relationship and marital problems, grief, variety of disorders |
| Smith et al., 2012  (RCT) | USA | 5 supervisors (doctoral level clinical psychologists) | 97 therapists (counsellors)  INT: N = 32, 59% female, age (m) = 45.9, experience (m) = 8.4  AC: N = 32, 66% female, age (m) = 42.9, experience (m) = 8.5  CG: N = 33, 70% female, age (m) = 42.9, experience (m) = 7.8 | 5 60 minutes sessions of supervision over 7 weeks  INT: Tele-conferencing Supervision (Live Supervision)  AC: Tape Review Supervision  CG: No supervision (practice sessions) | 5 counseling sessions over 7 weeks with standard patient scripts  Motivational Interviewing |
| Tanner et al., 2012  (controlled) | USA | 3 supervisors (licensed CBT-therapists) | 76 therapists (psychiatrists in training, clinical psychology PhD program), 91.1% female | Group supervision, additionally individual supervision  INT: Cotherapy Supervision  CG: Solo Therapy | 236 patients with various diagnoses, 61.7% female, age (m) = 36.19  INT: N = 30  CG: N = 206 (of which 146 with therapy by trainees who never received cotherapy supervision)  CBT-therapy |
| Weck et al., 2016  (RCT) | Germany | 9 supervisors (licensed clinical psychologists, licensed supervisors), age (m) = 43.0 | 23 therapists (psychotherapists in training, CBT)  INT: N = 11, 90.9% female  age (m) = 29.45  experience (m) = 2.64 y  CG: N = 12, 83.3% female  age (m) = 29.17  experience (m) = 2.35 y | 6 50-minutes supervision sessions (every fourth therapy session)  INT: Bug-in-the-eye-supervision (BITE; four BITE sessions, two DVB sessions)  CG: DVB Supervision | 42 patients with various diagnoses  INT: N = 19, 57.9% female,  age (m) = 36.16  CG: N = 23, 65.5% female  age (m) = 34.43  25 sessions of CBT-therapy |
| Weck et al., 2024  (RCT) | Germany | 6 supervisors (clinical psychologists, CBT, 1 day supervision workshop), 100% female, age (m) = 38.00 | 73 therapists (bachelor’s and master’s psychology students), 84.9% female, age (m) = 25.86 | 2 40-minutes supervision sessions  INT: Video-based supervision  CG: Verbal report-based supervision | Standardized patient with depression (played by 4 different students)  3 sessions of 20-minutes CBT-therapy |
| Wittenborn et al., 2022  (multiple baseline) | USA | 1 supervisor (licensed marriage and family therapist with expertise in emotionally focused therapy) | 4 therapists (2 doctoral‐level interns, 2 master's‐level social work students), 75% female, age (range) = 26- 32 | Video Feedback was added to emotionally focused therapy supervision | One focal couple treated with emotionally focused therapy |

*Note:* CBT = Cognitive Behavioral Therapy; exp. = experience; y = years; (m) = mean; ICBT = Internet-delivered CBT; INT = Intervention Group; CG = Control Group; SAU = Supervision As Usual; BITE = Bug-in-the-eye-supervision; DVB = Delayed Video Based; ^1^Only data from Phase 2 is included, since data from Phase 1 is included in Meza et al., 2023; ^2^There was another treatment arm, without training, which is excluded in this review; both treatment arms here included a training before supervision phase.

# Appendix E: Main outcomes of included studies

| **Study (Design)** | **Relevant outcomes (measures)** | **Main findings as reported in the publications** |
| --- | --- | --- |
| Alfonsson et al., 2020  (single-case experimental multiple baseline design) | CBT competence (CTS-R)  Supervisory Working Alliance (SWAI)  Satisfaction with supervision (SSQ-8) | - CBT competence is significantly higher in supervision phase than in baseline phase; supervision significantly improves therapists' CBT competence |
| Anderson et al., 2012  (uncontrolled) | Supervisory and therapeutic adherence (VTSS) | - Clinical supervision significantly influences therapist’s adherence to manualized techniques in TLDP - The most significant increase in adherence occurred during the intensive training year - Adherence levels decreased in the post training year, so effects of supervision may not be sustained over time without continued intensive training |
| Andersson et al., 2023  (controlled, factorial design 2x2x2x3) | Patients’ depressive symptoms (BDI-II; PHQ-9)  Patients’ anxiety (GAD-7)  Patients’ quality of life (BBQ)  Patients’ Insomnia (ISI) | - No significant difference between patients who have been discussed in supervision vs. patients who have not been discussed in supervision regarding any of the patient outcomes   (Note: Patients were randomized, not therapists, advice for one patient may be useful for another patient) |
| Bambling et al., 2006  (RCT) | Patients’ depressive symptoms (BDI)  Adherence (PST adherence scale)  Working alliance (WAI) | - Both supervision conditions showed significant improvements in patient-rated working alliance, greater symptom reduction of major depression, higher treatment retention rates, and more positive patient evaluations compared to the unsupervised condition. - No significant differences between the two supervision approaches (skill-focused vs. process-focused) in terms of their impact on working alliance or symptom reduction. - No correlation between PST-adherence and symptom reduction (*r* = .126; *p* = .163) or working alliance (*r* = .131; *p* = .322) |
| Bartle-Haring et al., 2009  (uncontrolled) | Primary Outcome: Progress on problem solving | - Live supervision did increase therapists’ perception of progress but not patients’ perception of progress - Therapists who received live supervision rated their patients' progress as improving more significantly over time compared to cases without live supervision. - Patients did not report similar improvements in their perception of progress |
| Bearman et al., 2017  (RCT) | Fidelity (TIEBI)  CBT expertise (Knowledge Test)  Global CBT competence (CBTCOMP-YD) | - Significant impact of the supervision condition on cognitive restructuring fidelity (*β =*.50), CBT expertise (*β* = 1.04) and global CBT competence (*β* = .32). |
| Beidas et al., 2012  (uncontrolled for supervision condition) | Adherence, Skill, Knowledge (Adherence and Skill Checklist, Knowledge Test) | - Number of supervision hours significantly predicted adherence (*β* = .39) and skill (*β* = .30) but did not significantly predict knowledge. |
| Bradshaw et al., 2007  (controlled) | Knowledge (Multiple Choice Test), Patients’ symptoms (KGV (M))  Patients’ social functioning (SFS) | - No significant differences in knowledge between the groups. - Significant larger symptom reduction in KGV scores in the supervision group. - No significant differences between groups for social functioning. |
| Carmel et al., 2016  (RCT) | DBT Skills and knowledge (exam), Supervision effectiveness (MCSS-26), therapist burnout (CBI) | - Marginally significant higher scores of BITE group regarding skills and knowledge. - No significant differences between supervision groups regarding supervision effectiveness and therapist burnout |
| Dorsey et al., 2025  (RCT)^1^ | TF-CBT adherence (TPOCS-S) | - No significant differences between supervision conditions on adherence of any phases of TF-CBT (stabilization, gradual exposure, trauma narrative) |
| Ellis et al., 2002  (“Study 2”; RCT) | Anxiety (STAI; Mahl’s Speech Disturbance Ratio)  Empathy (Referent and Manner scales of the Therapist Experiencing Scale) | - No significant differences between self-awareness conditions for any of the outcomes. |
| Johnson et al., 2023  (uncontrolled) | CBT competence (CTRS-CA)  Patients’ symptoms (PSC-17) | - Significant increase in competence at post-test - Significant improvements in patients’ symptom scores from pre- to post-test. |
| Karlsruher, 1976  (RCT) | Psychotherapeutic change (Child’s perspective: California test of personality, teacher’s perspective: Bristol Social Adjustment Guides, therapist’s perspective) | - Significant greater symptom reduction in supervised group than in non-supervised group regarding child’s and therapist’s perspective. - No significant difference regarding teacher’s perspective   (RCT also included a no-treatment group and a treated-by-professional-group: unsupervised non-professionals were similar to no-treatment control; symptoms in treated-by-professionals group generally closer to supervised non-professionals, but no significant difference) |
| Kivlighan et al., 1991  (controlled) | Working alliance (WAI)  Effect of therapy session (SEQ) | - Patients seen by therapists in live supervision reported stronger working alliance and perceived sessions as rougher than patients seen by therapists who received videotaped supervision. - Live supervision resulted in changes in performance compared to videotaped supervision. |
| Krall & Fürst, 2011  (uncontrolled) | Goal attainment and helpfulness of supervision (self-report questionnaires) | - Goals are attained and supervision is rated as helpful. - Scenic methods are rated as more helpful by the supervisor than by the supervisees. |
| Krall, 2022  (controlled) | Alliance in supervision group (questionnaire)  Goal attainment (questionnaire)  Helpfullness of supervision: Factors of supervision, feedback from the supervisor, transfer to psychotherapeutic work (questionnaire)  Workload of students (questionnaire) | - Goals are attained to a significantly higher extent in the psychodramatic and sociometric group (*d* = .49) - Significantly higher transfer to own practice (*d* = .54), alliance in supervision group (*d* = .87) and helpful rated feedback (*d* = .55) in psychodramatic and sociometric supervision - No significant difference in reduction of workload between supervision conditions |
| Maaß et al., 2024  (RCT) | CBT skills (CTS)  Communication skills (Clinical Communication Skills Scale – Short  Version; CCSS-S)  Therapeutic Alliance (Helping Alliance Questionnaire; HAQ) | - Students in live supervision group were rated significantly higher in competence than students in the control group. - No significant differences in the self-reported or patient-rated other outcomes. |
| Maaß et al., 2025  (RCT) | CBT skills (CTS)  Communication skills (CCSS-S)  Therapeutic Alliance (HAQ) | - Significant improvement of all outcomes in both conditions over time (*d* = 0.29 - *d* = 0.43) - No significant differences between supervision conditions for any of the outcomes (raters’ perspective; *d* = 0.001 – *d* = 0.19) |
| Martino et al., 2016  (RCT) | Adherence and competence in Motivational Interviewing (ITRS)  Patients’ treatment retention  Patients’ substance use (SUC) | - Significant greater improvement in competence in MIA: STEP supervision compared with SAU - No significant difference in adherence, in patients’ treatment retention and substance abstinence between the supervision conditions |
| Meza et al., 2023  (uncontrolled) | Observer-rated supervision techniques (SPOCS)  Observer-rated treatment delivery (TPOCS-S) | - Less intensive techniques were more commonly used in supervision sessions (session-level: 42.17% supportive–directive cluster, 57.83% supportive cluster) - clinicians receiving supportive-directive supervision were 18.46 more likely to deliver the trauma narrative than clinicians receiving supportive supervision |
| Monson et al., 2018  (RCT) | Patients’ PTSD symptoms (PCL)  Other patients’ outcomes (OQ-45) | - Significant difference in PCL-scores between supervision conditions. Largest symptom reductions in patients whose therapists received standard consultation (*d* = -1.78), then standard + audio consultation (*d* = -1.09) and smallest symptom reduction in no-consultation condition (*d* = -0.95); pairwise comparison revealed significant difference between standard consultation and no-consultation condition. - No significant difference in OQ-45 scores between conditions. |
| Ng & Cheung, 2007  (uncontrolled) | Declarative Knowledge about CBT for psychosis (case formulation test with 2 case vignettes)  Procedural skills (CTRS) | - Significantly more case formulations are rated ‘acceptable’ (good enough or good) post-supervision phase than pre-supervision phase - Significant improvements in supervisees competence from pre- to post-supervision phase |
| Ögren & Jonsson, 2003  (uncontrolled) | Psychotherapeutic Skills (MSES) | - Supervisees rated their overall psychotherapeutic skills significantly higher after supervision compared to before, most changes in the subscale psychodynamic understanding - Supervisors rated supervisees’ skills even higher than the supervisees’ themselves |
| Plantade-Gipch et al., 2021  (controlled) | Therapeutic alliance (WAI)  Supervisees’ emotion regulation (DERS) | - Significant difference in increase of therapeutic alliance in the supervision condition compared to the no-supervision condition (η^2^ = 0.05) - Therapists’ impulsiveness in the supervision increased significantly less than in the no-supervision condition |
| Rakovshik et al., 2016  (RCT) | CBT skills (CTS) | - Significant better CBT skills in the supervision condition compared to consultation sheet control group (*d* = 0.97). |
| Reese, Aldarondo, et al., 2009  (uncontrolled) | Satisfaction with supervision (SSQ)  Supervisory Working Alliance (SWAI-T)  Counsellors self-efficacy (COSE) | - No significant differences between video conferencing and in person rotation in satisfaction and supervisors working alliance. - Self-efficacy improved from pre- to posttest. |
| Reese, Usher, et al., 2009  (controlled) | Patients’ progress (ORS)  Therapeutic Alliance (SRS)  Supervisee’s satisfaction (SOS)  Supervisory Working Alliance (SWAI-T)  Counsellors self-efficacy (COSE) | - Better outcomes of patients in the feedback-condition than in the no-feedback condition (η^2 =^.07) - No significant difference in supervisory relationship, satisfaction with supervision or increases in counseling self-efficacy between feedback conditions. - quality of the supervisory alliance is related to the quality of the therapeutic alliance |
| Smith et al., (2012)  (RCT) | Motivational Interviewing Skill and Adherence (MITI) | - Significantly higher skill (spirit and empathy) and adherence rating in tele-conferencing supervision condition compared to no supervision (workshop only). - Marginally significant higher skill (spirit significant, empathy marginally significant) and adherence in tape review supervision condition compared to no supervision. - No significant difference in these outcomes between the two active supervision conditions. |
| Tanner et al., 2012  (controlled) | Patients’ functioning (OQ-45) | - No significant difference in patients’ functioning scores between supervision conditions - Improvements in functioning in both supervision conditions |
| Weck et al., 2016  (RCT) | Therapist competence (CTS)  Alliance (HAQ)  General Psychopathology (BSI)  Symptoms of depression (BDI-II) | - Significantly higher level of competence and alliance in the BITE than in the DVB condition - No significant differences in BSI or BDI-scores between supervision conditions |
| Weck et al., 2024  (RCT) | Therapists’ competence (CTS)  Therapists’ communication skills (CCSS-S)  Alliance (HAQ)  Empathy (ES) | - Significant improvement in rater-based competence, alliance, and empathy from pre to post assessment - All outcomes except empathy significantly lower at follow-up than at pre-assessment - No significant difference between two active supervision conditions for any of the outcomes |
| Wittenborn et al., 2022 | Therapists’ level of development (SLQ-R) | - Significant improvements in the professional security subscale for two out of three participants. - No significant improvements for SLQ-R score and professional self-confidence subscale, except for one participant. |

*Notes:* CBT = Cognitive Behavioral Therapy; CTS-R = Cognitive Therapy Rating Scale Revised; SWAI = Supervision Working Alliance Inventory; SSQ = Supervisee Satisfaction Questionnaire; VTSS = Vanderbilt Therapeutic Strategy Scale; TLDP = Time-limited dynamic psychotherapy; BDI = Beck Depression Inventory; PHQ = Patient Health Questionnaire; GAD = Generalized Anxiety Disorder; BBQ = Brunnsviken Brief Quality of Life Scale; ISI = Insomnia Severity Index; WAI = Working Alliance Inventory; TIEBI = Therapist Integrity to Evidence Based Interventions; CBTCOMP-YD = Manual for the Cognitive Behavioral Therapy Competence Observational Measure of Performance with Youth Depression; KGV (M) = Krawiecka, Goldberg and Vaughan symptom scale modified; SFS = Social Functioning Scale; MCSS-26 = Manchester Clinical Supervision Scale–26; CBI = Copenhagen Burnout Inventory; BITE = Bug-in-the-eye-supervision; TF-CBT = Trauma-Focused Cognitive Behavioral Therapy; TPOCS-S = Therapeutic Process Observational Coding System for Child Psychotherapy; STAI = State-Trait Anxiety Inventory; CTRS = Cognitive Therapy Rating Scale for Children and Adolescents PSC-17 = Pediatric symptom checklist-17 SEQ = Session Evaluation Questionnaire; CTS = Cognitive Therapy Scale; CCSS-S = Clinical Communication Skills Scale – Short Version; HAQ = Helping Alliance Questionnaire; ITRS = Independent Tape Rater Scale, MIA = Motivational Interviewing Assessment; STEP = Supervisory Tools for Enhancing Proficiency; SAU = Supervision As Usual; SUC = Substance Use Calendar; SPOCS = Supervision Process Observational Coding System; TPOCS-S = Therapeutic Process Observational Coding System for Child Psychotherapy; PCL = PTSD Checklist OQ-45 = Outcomes Questionnaire-45; CPT = Cognitive Processing Therapy CTRS = Cognitive Therapy Rating Scale; MSES = Modified Self Evaluation Scale; DERS = Difficulties in Emotion Regulation Scale; COSE = Counselling Self-Estimate Inventory; SRS = Supervision Rating Scale SOS = Supervision Outcome Survey; MITI = Motivational Interviewing Treatment Integrity; BSI = Brief Symptom Inventory; ES = Empathy Scale; SLQ-R = Supervisee Levels Questionnaire; ^1^Only data from Phase 2 is included, since data from Phase 1 is included in Meza et al., 2023.

# Appendix F: Risk of Bias Rating

**Risk of Bias rating of RCTs (ROB-2)**


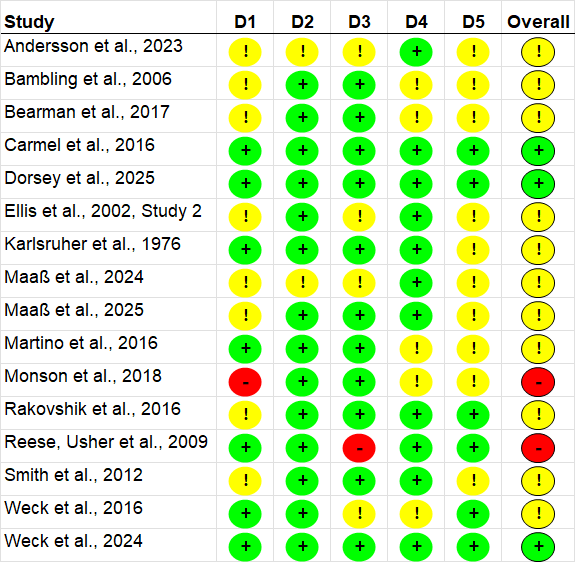


| **Domains** | |  |  |  |
| --- | --- | --- | --- | --- |
| D1 | Randomization process |  |  | Low Risk of Bias |
| D2 | Deviations from intended intervention |  | \|  \| \| --- \| | Some Concerns |
| D3 | Missing outcome data |  | \|  \| \| --- \| | High Risk of Bias |
| D4 | Measurement of the outcome |  |  |  |
| D5 | Selection of the reported results |  |  |  |

**Risk of Bias rating of controlled and uncontrolled trials (ROBINS-I V2)**


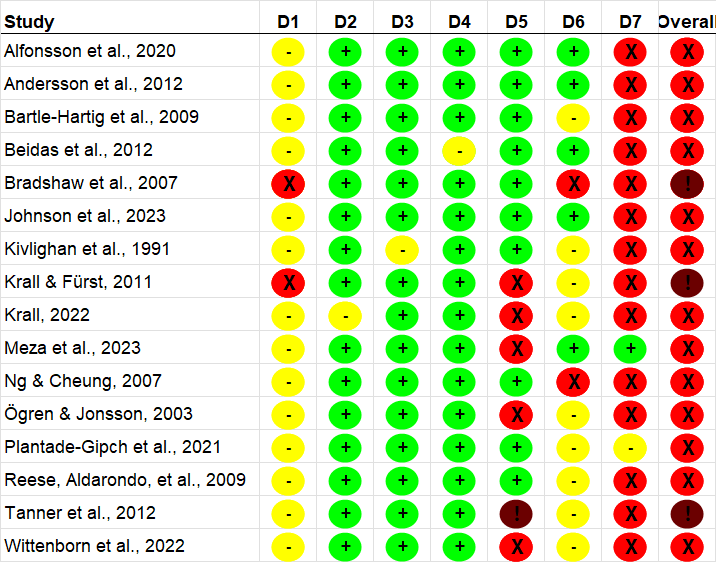


^1^

^2^

*Notes*: ^1^ Due to Serious risk of bias in several domains, the overall evaluation indicates a Critical risk of bias following the ROBINS algorithm. ^2^ Due to Moderate risk of bias in several domains, the overall evaluation indicates a Serious risk of bias following the ROBINS algorithm.

| **Domains** | |  |  |  |
| --- | --- | --- | --- | --- |
| D1 | Bias due to confounding |  |  | Low Risk of Bias |
| D2 | Bias in classification of interventions |  | \|  \| \| --- \| | Moderate Risk of Bias |
| D3 | Bias in selection of participants into the study (or into the analysis) |  |  | Serious Risk of Bias |
| D4 | Bias due to deviations from intended interventions |  |  | Critical Risk of Bias |
| D5 | Bias due to missing data |  |  |  |
| D6 | Bias in measurement of the outcome |  |  |  |
| D7 | Bias in selection of the reported result |  |  |  |

# Appendix G: GRADE Evidence Profile

| **Certainty assessment** | | | | | | | **№ of patients** | | **Effect** | | **Certainty** |
| --- | --- | --- | --- | --- | --- | --- | --- | --- | --- | --- | --- |
| **№ of studíes** | **Study Design** | **Risk of Bias** | **Inconsistency** | **Indirectness** | **Imprecision** | **Other factors** | **Intervention** | **Control** | **Relative (95% CI)** | **Absolute (95% CI)** |  |
| **Supervisees' competence** | | | | | | | | | | | |
| 7 | (randomized) controlled trial | serious | serious^a^ | not serious | serious^b^ | none | 188 | 187 | - | SMD **0.47** (-0.14 to 1.07) | ⨁◯◯◯ Very low^a,b^ |
| **Therapeutic alliance** | | | | | | | | | | | |
| 7 | (randomized) controlled trial | serious | serious^c^ | not serious | very serious^b,d^ | none | 225 | 225 | - | SMD **0.52**  (-0.23 to 1.27) | ⨁◯◯◯ Very low^b,c,d^ |
| **Clients’ symptoms** | | | | | | | | | | | |
| 7 | (randomized) controlled trial | serious | serious ^e^ | not serious | serious^b^ | none | 611 | 655 | - | SMD -**0.24**  (-0.48 to 0) | ⨁◯◯◯ Very low^b,e^ |

**CI:** confidence interval; **SMD:** standardised mean difference

**Explanations**

a. Substantial heterogeneity 79.10%

b. Confidence interval includes zero.

c. Substantial heterogeneity 88.98%

d. Wide confidence interval.

e. Substantial heterogeneity 65.89%
